# Integrated genome-wide domestication and association analyses reveal the complex genetic basis of parthenocarpy during cucumber domestication

**DOI:** 10.1186/s43897-025-00182-y

**Published:** 2026-01-07

**Authors:** Pinyu Zhu, Shiyou Wang, Yongjiao Meng, Weiping Diao, Xiaqing Yu, Yuhui Wang, Ji Li, Jinfeng Chen

**Affiliations:** 1 State Key Laboratory of Crop Genetics & Germplasm Enhancement and Utilization, College of Horticulture, Nanjing Agricultural University, Weigang Campus, Nanjing, 210095 China; 2 Jiangsu Key Laboratory for Horticultural Crop Genetic Improvement, Institute of Vegetable Crops, Jiangsu Academy of Agricultural Sciences, Nanjing, 210014 China

**Keywords:** Parthenocarpy, Cucumber, Genetic basis, GWAS, Domestication

## Abstract

Parthenocarpy is an important target trait for the yield and quality of fruit crops. It has been used and selected in cucumber breeding for more than a hundred years, which has led to variation in parthenocarpic ability among different cucumber groups and thus resulted in a complex genetic basis. Here, we performed deep resequencing of 236 cucumbers from six cucumber groups and investigated parthenocarpic phenotypes over three years and six seasons to explore the genetic basis of parthenocarpy in cucumber. Among the 34 loci associated with parthenocarpy identified through a genome-wide association study (GWAS), 17 (50%) were correlated with reported quantitative trait loci (QTLs), and 27 (79.4%) were selected during cucumber domestication. We found that favorable parthenocarpic alleles continuously accumulated in cultivated cucumbers, and our data pinpoint that different favorable alleles were selected in the cucumber groups, leading to variations in parthenocarpic ability among them. To validate the favorable alleles detected by GWAS, we cloned three genes, *CsACA10*, *CsCaM*, and *CsERT2*, and verified their roles in the regulation of parthenocarpy for the first time. Our study elucidated the genetic basis and molecular mechanism of parthenocarpy, providing important germplasms for improving parthenocarpy in cucumber breeding.

**Supplementary Information:**

The online version contains supplementary material available at 10.1186/s43897-025-00182-y.

## Core

The complex genetic basis of parthenocarpy during domestication was revealed through genome-wide domestication and association analyses. Variation in parthenocarpic ability among different cucumber groups is regulated mainly through the selection of different parthenocarpic-related loci. Three genes, *CsACA10*, *CsCaM*, and *CsERT2*, were subsequently cloned from parthenocarpic loci, and their roles in the regulation of parthenocarpy were verified.

## Gene & accession numbers

The sequence data of cucumber from this study can be found in the genome database of 9930 V3.0 (http://cucurbitgenomics.org/organism/20), including *CsaV3_1G037120*, *CsaV3_1G037130*, *CsaV3_1G037140* (*CsERT2*), *CsaV3_1G037150*, *CsaV3_1G037160*, *CsaV3_3G032560* (*CsACA10*), *CsaV3_3G032570*, *CsaV3_3G032540*, *CsaV3_3G032550*, *CsaV3_3G032560* (*CsCaM*), *CsaV3_3G032570*, *CsaV3_3G032580*, and *CsaV3_3G032590*. The depth-resequencing data can be found in the BioProject database of the National Center for Biotechnology Information under the accessions PRJNA1035551 and PRJNA1031793.

## Introduction

Fruit development is normally dependent on successful pollination and fertilization in plants. However, pollination and fertilization can easily be hindered by environmental conditions such as high temperature, high humidity, and a lack of pollinators. In some angiosperms, the development of fruit can occur independently of pollination and fertilization, a phenomenon known as parthenocarpy (Gustafson et al. 1942). In commercial production, parthenocarpy has been recognized as a highly attractive agronomic trait to increase the yield of fruit crops in undesirable environments (Li et al. 2014, Sun et al. 2016; Matsuo et al. 2020). The production of parthenocarpic fruits is an effective approach for fruit crops with undesirable large or hard seeds (Fabrice et al. 2000). Moreover, parthenocarpic fruits can also improve the fruit quality of crops, including increasing the shelf life of tomatoes, increasing the edible content of cucumber, and preventing browning and bitterness caused by the seeds of eggplants (Pandolfini et al. 2009; Klap et al. 2017; Nie et al. 2025).

Cucumber (*Cucumis sativus* L.) is a major vegetable crop cultivated worldwide and was domesticated from wild cucumber (*C*. *sativus* L. *var*. *hardwickii*) in Asia approximately 3000 years ago (Whitaker and Davis 1962; Yang et al. 2012). During the initial domestication of cucumber, breeders and farmers selected for target traits to meet human consumption needs, resulting in cultivated cucumbers with extreme phenotypic variation in fruit shape, size, spines, warts, sex types, bitterness, flower time, etc. (Qi et al. 2013; Zhang et al. 2015; Gao et al. 2020; Pan et al. 2020; Wang et al. 2020; Li et al. 2022a; Zhu et al. 2022). Unlike these domestication-related traits, parthenocarpy may not have been a main target trait during the initial domestication of cucumbers. Most cucumber varieties do not exhibit parthenocarpy in the initial stages of open field cultivation. In the early twentieth century, breeders and famers conducted artificial domestication for parthenocarpy in Europe. The parthenocarpic varieties were subsequently selected and applied in the production of greenhouse cucumbers to increase fruit yield (Strong 1921; Wellington and Hawthorn 1928). Additionally, the parthenocarpy trait from European cucumbers has been utilized to mitigate the issue of first fruit inhibition in U.S. processing cucumbers (Pike and Peterson, 1969; De Ponti 1976; El-Shawaf and Baker 1981). Throughout the course of East Asian cucumber breeding, the parthenocarpy trait has also been selected for cucumber breeding (Feng et al. 2020). As a result of parthenocarpic domestication, cultivated cucumber exhibited extreme variations in parthenocarpic ability among different cucumber groups.

Parthenocarpy is a complex trait resulting from interactions between genotype and the environment. Early genetic studies on cucumber have indicated that parthenocarpy is controlled by single-gene inheritance (Pike and Peterson, 1969; De Ponti et al. 1976). However, recent studies have generally revealed that parthenocarpy is a complex quantitative trait, and at least 15 QTLs with diverse genetic backgrounds have been identified using traditional QTL mapping or bulk segregant analysis sequencing (Wu et al. 2016; Lietzow et al. 2016; He et al. 2019; Niu et al. 2020; Zhao et al. 2022; Devi et al. 2022). For example, Lietzow et al. (2016) identified seven QTLs associated with parthenocarpy on chromosomes 2, 4, 5, 6, and 7 in North American processing cucumber (Lietzow et al. 2016). Wu et al. (2016) detected a major-effect QTL, Parth2.1, and six minor-effect QTLs on chromosomes 1, 2, 3, 5, and 7 in European greenhouse cucumber (Wu et al. 2016). Niu et al. (2020) detected four novel parthenocarpy QTLs associated with South China cucumber on chromosomes 1, 3, and 6. To date, almost all QTLs have not been cloned because the complex genetic background of cucumber and limited recombination events make it difficult to estimate these positions of QTLs precisely. These studies revealed the genetic basis of parthenocarpy in certain cucumbers, but the genetic basis of parthenocarpy in natural populations remains largely unknown. Furthermore, whether these parthenocarpy-related genes/QTLs have contributed to parthenocarpy during domestication remains unclear.

The dissection of parthenocarpy response genes is a promising approach for enhancing parthenocarpic ability. Many parthenocarpy-related genes have been identified through map-based cloning or transgenic technology in Solanaceae crops, revealing that hormone signal transduction (Wang et al. 2005; Goetz et al. 2006; De Jong et al. 2011; Carrera et al. 2012; Mounet et al. 2012; Liu et al. 2018; Matsuo et al. 2020), calcium signal transduction (Wu et al. 2011), and MADS box-related genes (Pnueli et al. 1994; Vrebalov et al. 2009; Geuten et al. 2010; Huang et al. 2017; Ribelles et al. 2019; Okabe et al. 2019) contribute to the initiation of parthenocarpic fruit growth. In cucumber, only several auxin-related genes, such as the auxin receptor genes *CsTIR1* and *CsAFB2* (Xu et al. 2017), the auxin response factor *CsARF10* (Xu et al. 2024a, b), and APETALA2/ethylene responsive factor (AP2/ERF) family transcription factor *CsNPF1* (Nie et al. 2025), have been verified to be involved in the regulation of parthenocarpy through heterologous expression technology. However, these genes are not located in the regions of QTLs related to parthenocarpy.

GWAS is an effective tool for elucidating the genetic basis of complex traits in horticultural crops. For example, Qi et al. (2013) first constructed a genetic variation map of cucumber via whole-genome sequencing of 115 cucumber accessions. Many GWASs have been performed on 115 cucumber accessions to identify genomic regions associated with traits such as fruit bitterness (Shang et al. 2014), cotyledon regeneration (Wang et al. 2018a), hypocotyl length (Cai et al. 2020), and adult heat tolerance (Wang et al. 2023). Wang et al. (2018b) conducted a GWAS using 1234 cucumber lines and identified genomic regions significantly associated with 13 horticulturally important traits of cucumber, including fruit yield-related traits, disease resistance-related traits, and fruit shelf life-related traits. These studies demonstrate the power and credibility of GWASs. However, the functions of candidate genes within the associated regions identified by GWASs usually remain unknown. Cross-validation through the integration of multiomics approaches and transgenic technologies with GWASs will contribute to enhancing the accuracy of selecting candidate genes associated with GWASs.

In this study, we used a new association mapping panel of 236 cucumber inbred lines to elucidate the genetic basis of parthenocarpy by combining GWAS and genome-wide domestication analyses. The reliability of the GWAS results was validated via QTL mapping. Furthermore, we used analyses of gene expression, sequences, haplotypes, virus-induced gene silencing (VIGS), and protein interaction assays to validate the molecular mechanisms of three candidate genes that regulate cucumber parthenocarpy. These results provide valuable insight into the genetic basis and molecular mechanism of parthenocarpy in cucumber and other cucurbit crops.

## Results

### Population structure of 236 cucumber inbred lines

**Fig. 1:**
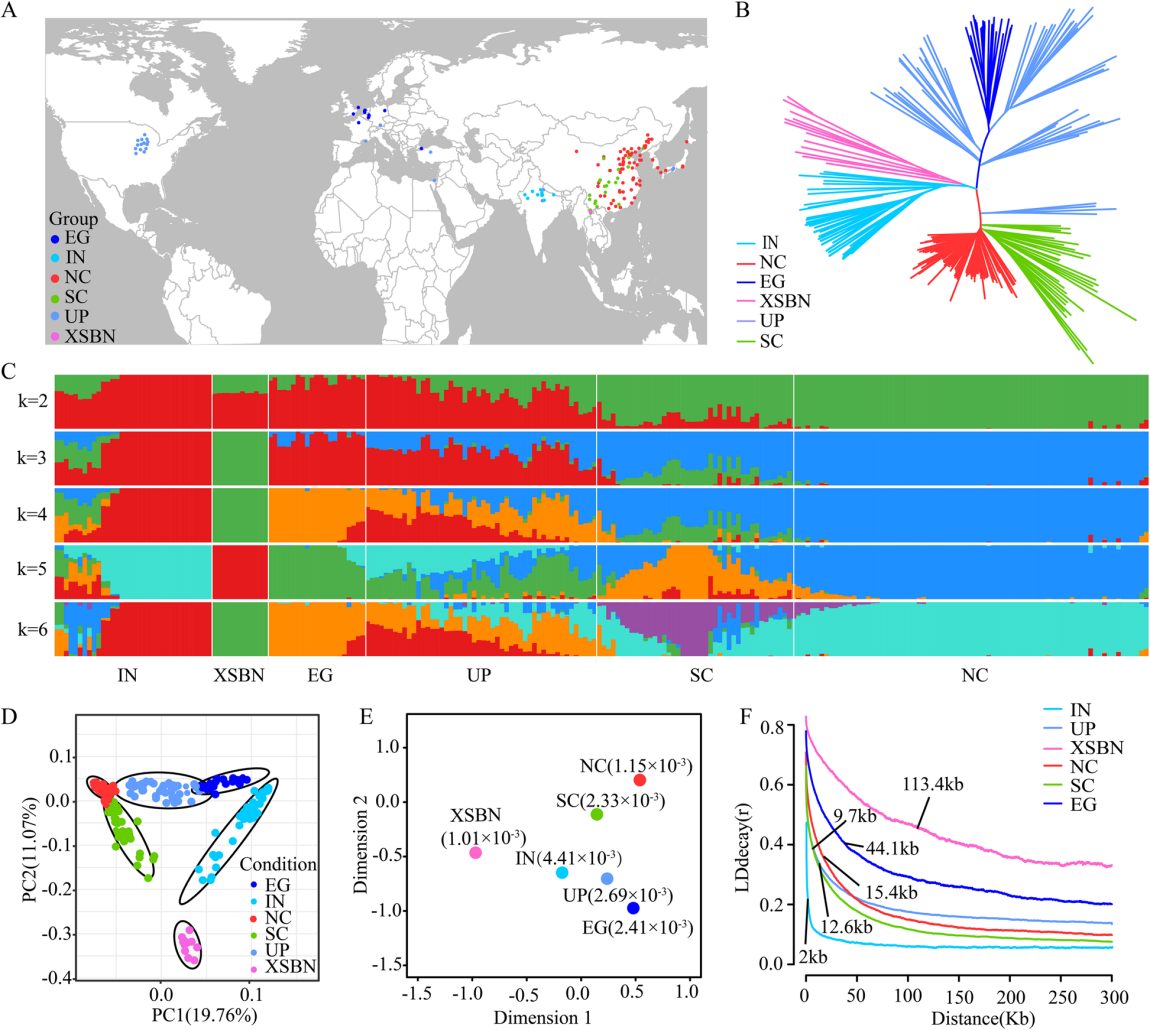
Geographic distribution and population structure of 236 cucumber inbred lines. **A** Geographic origin of 236 cucumber inbred lines generated via the ‘ggmap’ function from the R package. The colors indicate different groups: IN (sky blue), XSBN (pink), UP (teal), EG (blue), NC (red), and SC (green), as shown in **B**, **D**, **E**, and **F**. **B** Neighbor‒joining tree of 236 cucumber inbred lines. **C** ADMIXTURE model-based clustering analysis of 236 cucumber lines generated via fastSTRUCTURE, with the number of ancestry kinships ranging from 2 to 6. The Y-axis represents the number of cucumber groups (IN, red and mix color of blue, orange, etc.; XSBN, green; EG, orange; UP, mix color of red and orange, SC, mix color purple and teal; NC, teal), and the X-axis represents different cucumber inbred lines. **D** PCA plots of the population structure using the first two components. **E** Summary of the nucleotide diversity of cucumber groups and multidimensional scaling of pairwise F_ST_ values between different cucumber groups. **F** LD decay of the six cucumber groups. The LD distance decays to half the maximum LD decay

A previous study revealed that the population structure of cucumber includes one wild cucumber group (India cucumbers, IN) and three cultivated cucumber groups, Eurasian (EU), Xishuangbanna (XSBN), and East Asian (EA) cucumbers (Qi et al. 2013). Over the past decade, the increased frequency of gene flow among different cucumber groups may have led to a more complex population structure. Here, a mapping association panel of 236 cucumber inbred lines was constructed for high-depth resequencing (Fig. 1A), and a total of 2.1 Tb of raw data was obtained, with an average depth of 47 × and 97.8% genome coverage (Table S1). By mapping the sequencing reads to the 9930 genomes V3.0, we identified a total of 9,765,872 high-quality single-nucleotide polymorphisms (SNPs) after retaining the raw data. The population structure of the 236 cucumber inbred lines was inferred using ADMIXTURE, and a maximum likelihood (ML) tree was constructed on the basis of 1,283,903 SNPs (Figs. 1B and C). The population structure analysis of 236 cucumber inbred lines confirmed that K = 6 represented the best model for diverging the six distinct groups referred to as India (IN), U.S. processed (UP), European greenhouse (EG), North China (NC), South China (SC), and XSBN cucumbers (Fig. 1C, Fig. S2A). These results were further supported by principal component analysis (PCA) (Fig. 1D, Fig. S2B, Table S2) and multidimensional scaling of pairwise fixation index (F_ST_) values (Fig. 1E, Table S3). To elucidate the population structure of 236 cucumber inbred lines, we integrated the resequencing data of 236 cucumber inbred lines with 115 accessions from Qi et al. (2013). The phylogenetic clustering analysis revealed that: IN and XSBN groups co-clustered with the previously reported IN, XSBN groups; NC and SC groups grouped with the EA groups, and EG and UP cucumbers aligned with the EU groups ( Figs. S2B and S 2 C). Population differentiation analysis further supported these findings, with low pairwise F_ST_ value between corresponding four typically cucumber groups from both studies (IN: 0.0198, EA: 0.0231, EU: 0.00929, and XSBN: 0.0047). Hence, the phylogenetic structure of 236 cucumber lines was consistent with the phylogenetic structure of the 115 cucumber lines, and the high-depth resequencing of germplasm resources reported here serves as an important complementary resource for cucumber.

**Fig. 2:**
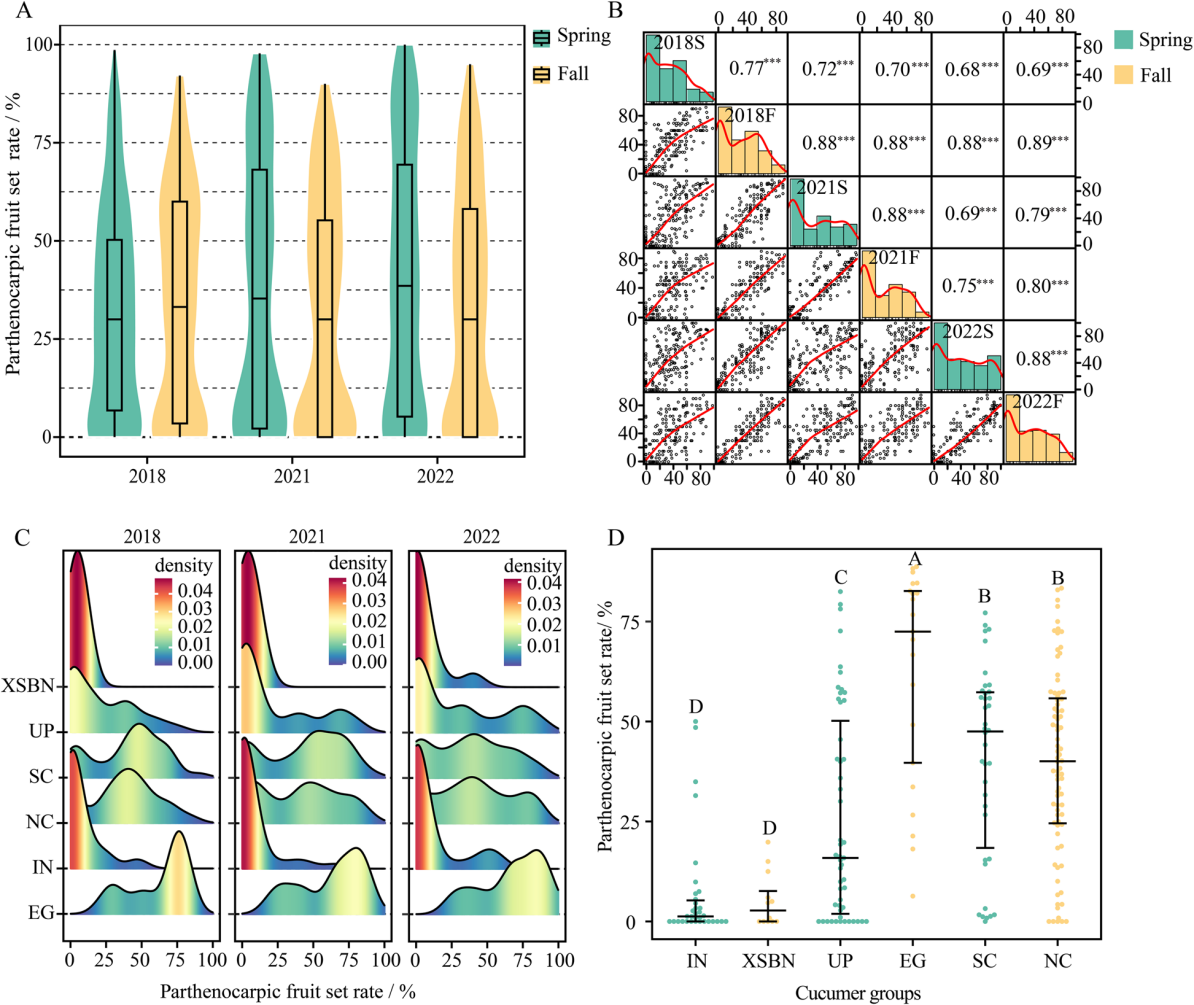
Phenotypic data analysis for parthenocarpy in 236 cucumber inbred lines based on the PFS rate across six environments: spring 2018 (2018S), fall 2018 (2018F), spring 2021 (2021S), fall 2021 (2021F), spring 2022 (2022S), and fall 2022 (2022F). **A** Violin and box plots depicting the distribution of the PFS rate across the six environments. **B** Distribution and correlation matrix of the PFS ratio across the six environments. *** indicates *p* < 0.001. **C** Density plot depicting the distribution of the PFS rate among the six cucumber groups in 2021, 2021, and 2022. **D** PFS rates of the six cucumber groups based on the mean values across all the environments. Different letters indicate *p* < 0.01, as determined via the Wilcoxon test

The genome-wide genetic diversity (nucleotide diversity, π) and linkage disequilibrium (LD) decay for each cucumber group were calculated. The π value of the IN group was greater than that of the other cucumber groups (Fig. 1E, Fig. S2D). Specifically, the average π values are 4.41 × 10^–3^, 1.01 × 10^–3^, 2.69 × 10^–3^, 2.41 × 10^–3^, 2.33 × 10^–3^, and 1.15 × 10^–3^ in the IN, XSBN, UP, EG, SC, and NC groups, respectively. This finding is consistent with India being the center of origin of cultivated cucumber (Qi et al. 2013; Wang et al. 2018b). A greater LD distance was observed in the cultivated groups (9.7 to 113.4 kb, Fig. 1F) than in the IN group (2 kb, Fig. 1F), suggesting that artificial selection played an important role in the domestication of cucumber.

### Parthenocarpic ability of the cucumber groups across six environments

Six environment trials were conducted to evaluate the parthenocarpic ability of 236 cucumber inbred lines. The phenotypic data of parthenocarpy revealed extreme phenotypic variation in the 236 cucumber inbred lines, with parthenocarpic fruit set (PFS) rates ranging from 0 to 100% (Fig. 2A). The distribution of the PFS rate for the 236 cucumber inbred lines across the 6 environment trials exhibited a clear bimodal distribution (Fig. 2A). The trends in the frequency distribution of the PFS rate were similar between any two environments, with significant positive correlations (*r* > 0.7) (Fig. 2B and C). Analysis of variance for parthenocarpy indicated that the genotype effect was the dominant component of variance, and the broad-sense heritability (*h*^2^) of parthenocarpy was 0.88 ( Table S4). The best linear unbiased prediction (BLUP) value of PFS was calculated for further GWAS. In addition, we observed that the parthenocarpic ability of cultivated cucumber groups (except for XSBN) was significantly greater than that of the IN cucumber group (Fig. 2C and D, Table S4), suggesting that the parthenocarpy trait is associated with domestication practices.

### GWAS for parthenocarpy through GEMMA and IIIVmrMLM

**Fig. 3:**
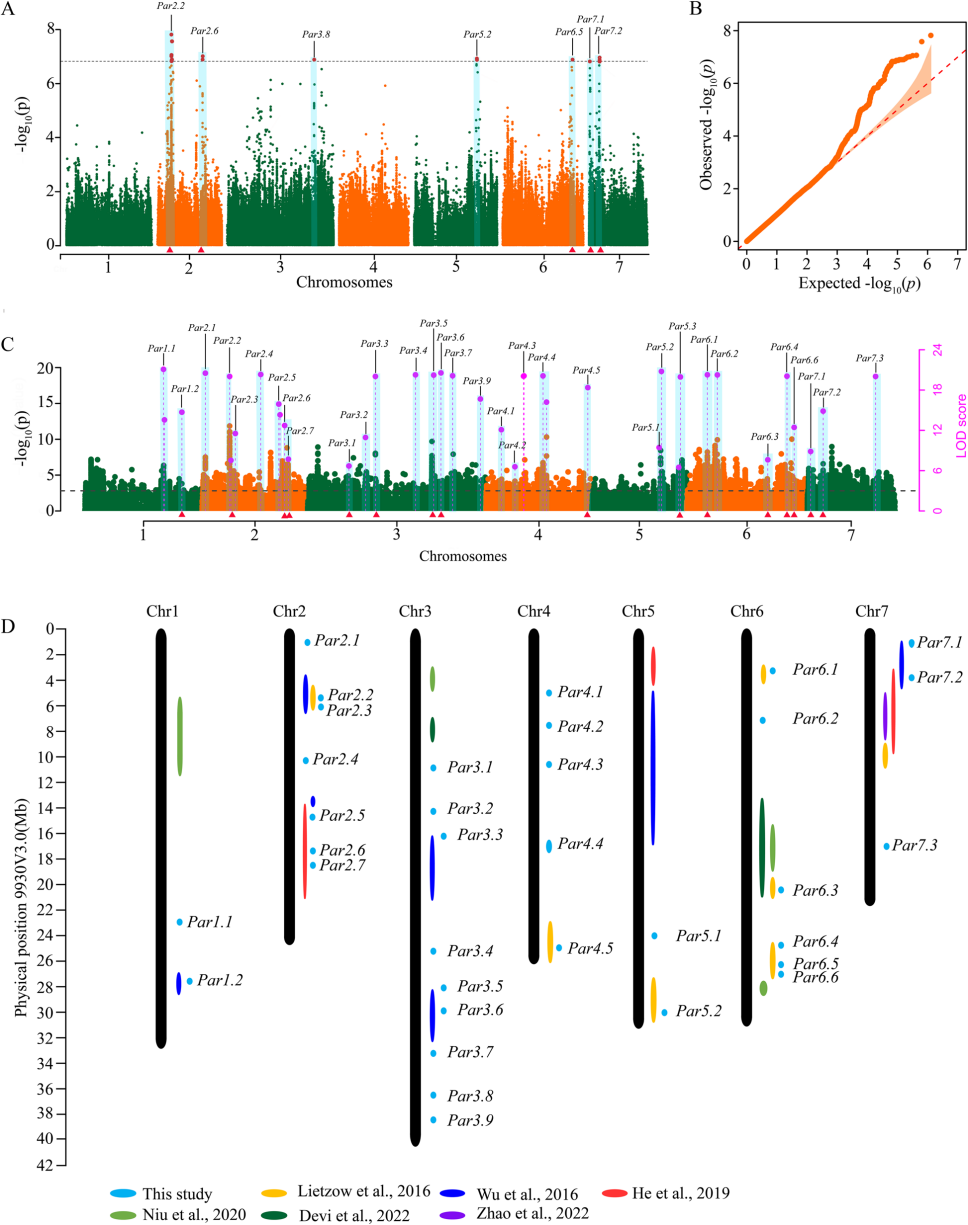
GWAS signals of parthenocarpy via the GEMMA and IIIVmrMLM methods. **A** Manhattan plots of GWAS results for parthenocarpy via the GEMMA method. The dashed line indicates the significance thresholds of the GEMMA method with *p* = 1.5 × 10^–7^. The red triangle at the bottom of each plot indicates that GWAS signals overlapped with reported QTLs in cucumber, as shown in **C**. **B** Q‒Q plots describing the distribution of the expected *p* value and the observed value via the GEMMA method. **C** Manhattan plots of the GWAS results via the IIIVmrMLM method. The Y-axis on the left side indicates the -log_10_ (*p* values) of the QEIs identified from single-marker genome-wide scanning in the first step of IIIVmrMLM, while the Y-axis on the right side represents the LOD value of the QTNs at the second step of IIIVmrMLM, with a threshold of LOD value = 3.0 (black dashed line). **D** Chromosomal locations of parthenocarpic QTL or GWAS signals in cucumber based on the physical location of the 9930 genome V3.0

A GWAS was performed to determine the genetic basis of parthenocarpy through the GEMMA and IIIVmrMLM methods. We identified seven GWAS signals for parthenocarpy via the GEMMA method (Fig. 3A and B, Table S6), with peak quantitative trait nucleotide (QTN) regions of *Par2.2* at 5.24 Mb, *Par2.6* at 17.43 Mb on chromosome 2, *Par3.8* at 33.40 Mb on chromosome 3, *Par5.2* at 23.96 Mb on chromosome 5, *Par6.5* at 26.71 Mb on chromosome 6, *Par7.1* at 0.38 Mb on chromosome 7, and *Par7.2* at 3.97 Mb on chromosome 7. The phenotypic variance explained (PVE) of these QTNs ranged from 2.70 to 7.49% ( Table S6). A total of 38 GWAS signals were detected via the IIIVmrMLM method, with LOD scores for each GWAS signal ranging from 6.5 to 129.9 and PVEs for each GWAS signal ranging from 0.36 to 4.66% ( Table S6).

We merged these significant GWAS signals from the GEMMA and IIIVmrMLM methods and identified a total of 34 GWAS signals (Fig. 3D, Table S6). Among them, five common GWAS signals (*Par2.2*, *Par2.6*, *Par5.2*, *Par7.1*, *Par7.2*) were identified through the two methods (Figs. 3A and B). A comparison of the regions of these GWAS signals with known parthenocarpy-related genes/QTLs in cucumber revealed that 17 GWAS signals were colocalized with parthenocarpy-related genes/QTLs (Fig. 3D, Tables S5 and S6), whereas 17 GWAS signals represented novel QTLs associated with parthenocarpy (Fig. 3D). By searching for candidate genes within a 50 kb region of the peak QTNs, we identified a total of 286 candidate genes for parthenocarpy ( Table S7). Among these candidate genes for parthenocarpy, we focused on those genes involved in the development of parthenocarpy in plants and identified 12 hormone-related genes, four calcium signal-related genes, one MADS-related gene, and one polyamine-related gene ( Table S8).

### Genetic basis of parthenocarpy during cucumber domestication

**Fig. 4:**
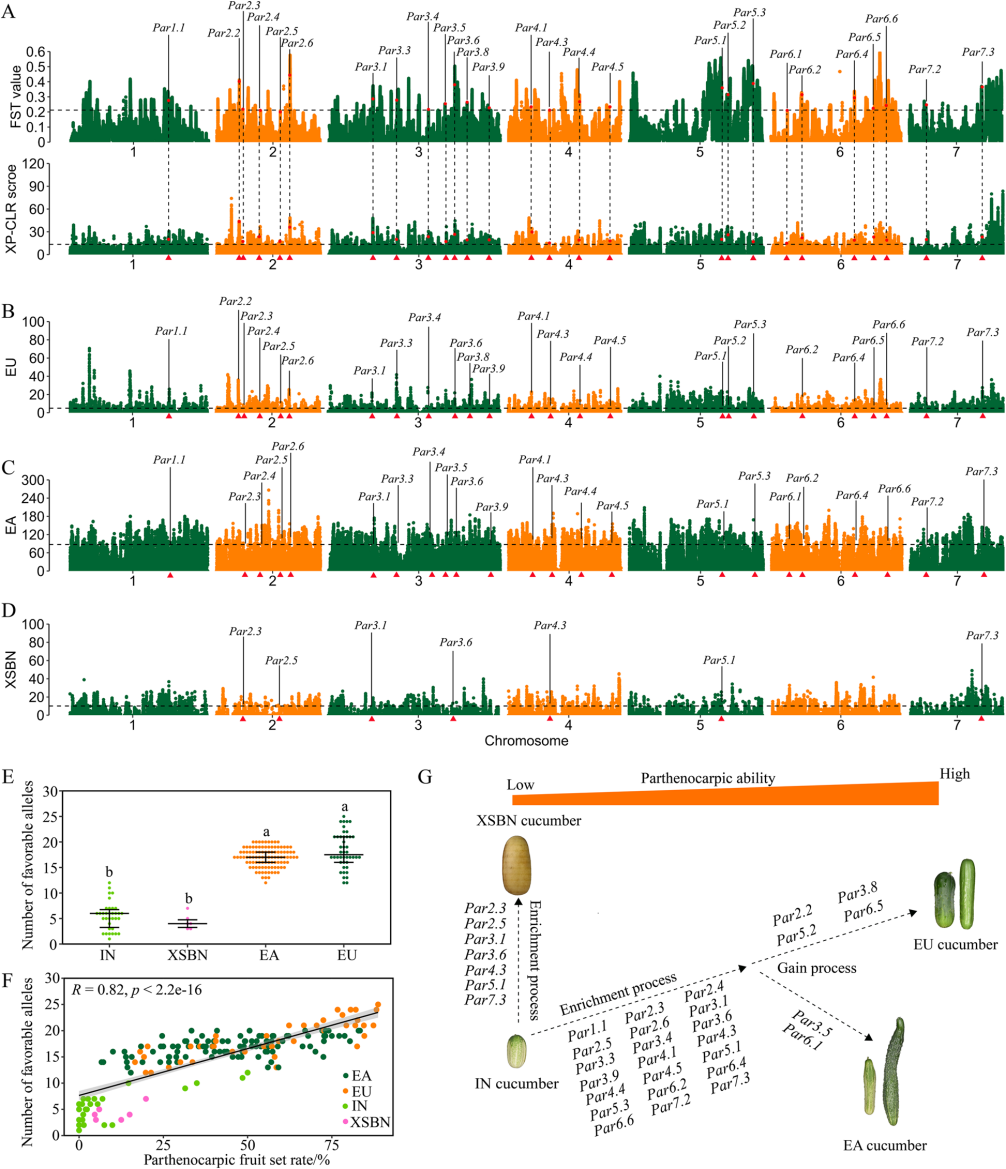
Genetic basis of parthenocarpy during cucumber domestication. **A** Genome-wide sweep detection via the F_ST_ method (top) and XP-CLR method (bottom). The horizontal line represents the threshold of the top 5% of the XP-CLR and F_ST_ values. The GWAS signals are shown in black text, and the red triangle at the bottom of each plot indicates that GWAS signal regions overlapped with selective sweeps in **A**, **B**, **C**, and **D**. Genome-wide selection signals in the EU group (**B**), EA group (**C**), and XSBN group (**D**) compared with IN groups via the XP-CLR method. The black lines represent the top 5% of the XP-CLR scores. **E** The number of favorable alleles related to parthenocarpy in different cucumber groups. In the cultivated cucumber groups, only cucumbers with parthenocarpic ability (PFS > 5%) were selected for data analysis, as shown in **F**. Different letters represent *p* < 0.05, as determined via the Wilcoxon test. **F** The number of favorable alleles corresponding to their PFS in cucumber groups. The *x*- and *y*-axes indicate the PFS rate and number of favorable alleles related to GWAS signals, respectively. Linear regression was used for the relevance analysis between the number of favorable alleles and the PFS rate. **G** Schematic of the two-step evolution of parthenocarpy in cucumber. Only GWAS signals within the putatively selected region are listed in this study. The enrichment process involves the accumulation of favorable alleles from IN cucumbers during XSBN, EA, and EU cucumber domestication. Base on the enrichment process, the gain process represents the acquisition of new favorable alleles in EA and EU cucumber domestication

To identify potential domestication sweeps related to parthenocarpy, the 134 highly parthenocarpic cultivated cucumbers were compared with 34 IN cucumbers using the cross-population composite likelihood ratio (XP-CLR) and fixation index (F_ST_) methods. We identified 131 selective sweeps related to cucumber domestication of parthenocarpy ( Table S9). A comparison of the physical positions of the GWAS signals and selective sweeps revealed that 27 GWAS signals overlapped with the 131 identified selective sweeps (Fig. 4A, Table S9), suggesting that these GWAS signals underwent selection during cucumber domestication. Based on the genetic flow of parthenocarpy between SC/NC and UP/EG groups, the classical four-groups was used for domestication analysis of cultivated groups. We also searched for the selective sweeps associated with three cultivated groups, EU (Fig. 4B), EA (Fig. 4C), and XSBN (Fig. 4D). By comparing the selective sweeps of three cultivated cucumber groups and GWAS signals of parthenocarpy, 25, 23, and 7 GWAS signals were directly selected during the domestication of EU, EA, and XSBN, respectively. Interestingly, 21 GWAS signals were commonly selected under domestication of EU and EA cucumbers (Fig. 4B, C), whereas four GWAS signals (*Par2.2*, *Par3.8*, *Par5.2*, and *Par6.5*) were uniquely selected in EU cucumbers (Fig. 4B), and two GWAS signals (*Par3.5*, and *Par6.1*) were uniquely selected in EA cucumbers (Fig. 4C).

To test for evidence of selection on parthenocarpic ability among 236 cucumber inbred lines, we summarized the frequency and number of favorable alleles on the basis of significant QTNs of 27 GWAS signals (Figs. 4E and 4 F; Fig. S4; Table S11). Compared with that in the IN groups, the frequency of favorable parthenocarpic alleles in the cultivated groups increased from 7.9 to 72.8%, indicating positive selection for favorable alleles during domestication ( Fig. S4). Of the 27 favorable alleles, 22 were selected from India cucumber with low to moderate frequencies, and 5 were gained from EU and EA cucumber ( Fig. S4). These favorable alleles in IN cucumbers are scattered across diverse cucumbers, with a total number ranging from 2 to 12 (Fig. 4E; Table S11). Among cultivated cucumbers, EU cucumbers possess a greater number of favorable alleles (ranging from 9 to 25, with an average of 18.2) compared to EA (ranging from 9 to 20, with an average of 16.3) and XSBN (ranging from 3 to 7, with an average of 4.3) cucumbers. Furthermore, the higher parthenocarpic cucumbers tended to have a greater number of favorable alleles among cultivated cucumbers (Fig. 4F). On the basis of these results, we also proposed a domestication process for parthenocarpy that emphasizes the continuous accumulation of favorable alleles associated with parthenocarpy in cultivated cucumbers through enrichment and gain process (Fig. 4G), resulting in significant phenotypic variation in parthenocarpic ability in cucumber.

### Validation of GWAS signals and selective sweeps using QTL mapping

**Fig. 5:**
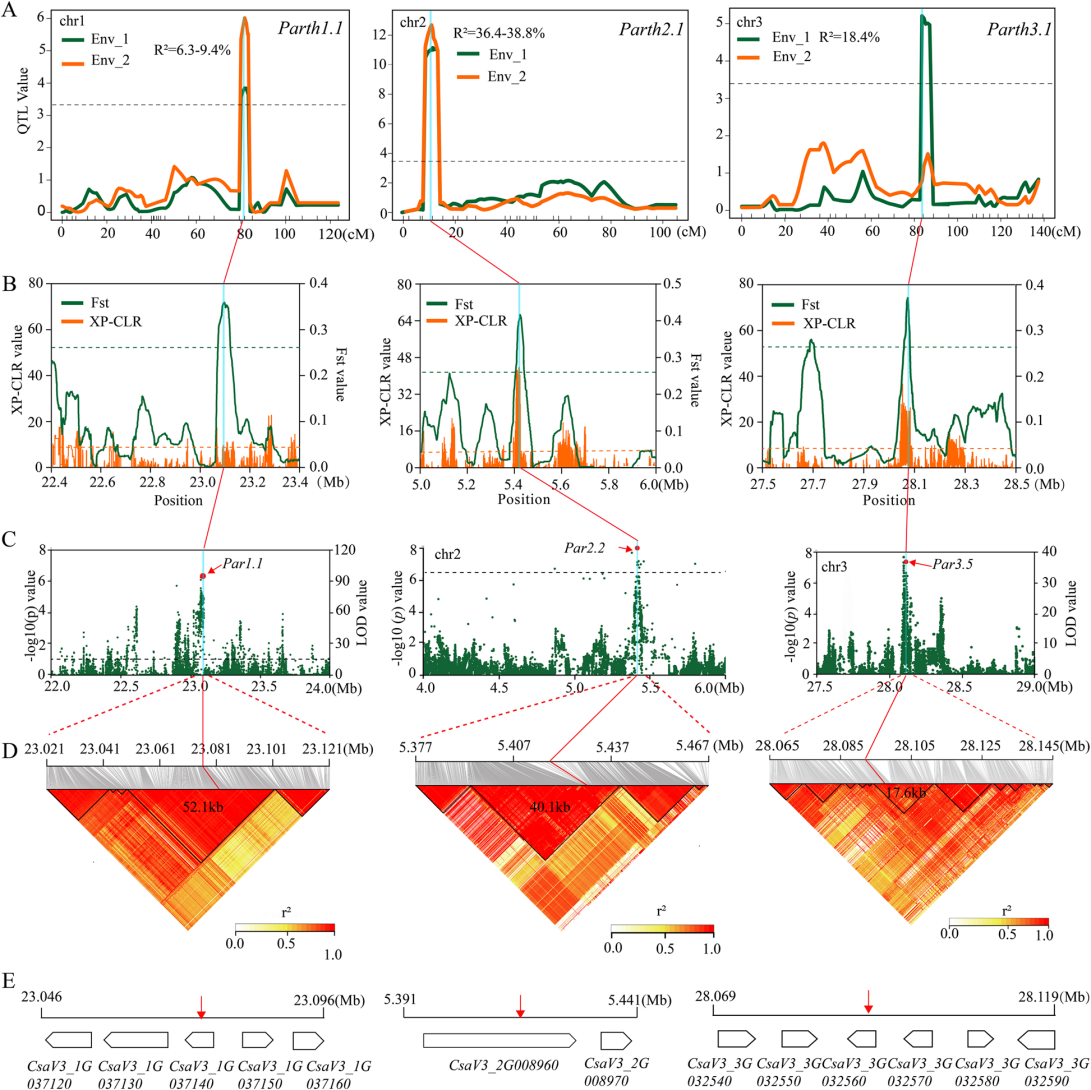
Candidate genes associated with three GWAS signals based on the analysis of genome-wide domestication and QTL mapping. **A** Three QTLs associated with parthenocarpy, *Parth1.1*, *Parth2.1*, and *Parth3.1*, were detected via QTL mapping of the F_2:3_ population derived from the cross between PE11 and PE12. The blue column represents peak regions of GWAS signals or selective sweeps, consistent with those shown in panels **B** and **C**. **B** The detected selective sweeps overlapped with *Parth1.1* (left), *Parth2.1* (middle), and *Parth3.1* (right). The peak regions of the Manhattan plots (**C**) and the LD heatmap (**D**) for GWAS signals *Par1.2* (left), *Par2.2* (middle), and *Par3.5* (right). **E** Annotated genes within the 50 kb region of *Par1.2* (left), *Par2.2* (middle), and *Par3.5* (right)

To validate the GWAS signals and selective sweeps that could be identified via QTL mapping, we constructed a genetic map (Table S12) and performed QTL mapping to identify parthenocarpy-associated regions based on an F_2:3_ population derived from the cross of PE11 and PE12. The distribution of the parthenocarpic fruit set rate was similar for the two experiments and showed a bimodal distribution (Fig. S5). Three QTLs, *Parth1.1*, *Parth2.1*, and *Parth3.1*, explaining 6.3% to 36.4% of the phenotypic variation, were identified on chromosomes 1, 2, and 3, respectively (Fig. 5A, Fig. S6; Table S13). We also observed that at least one selective sweep overlapped with the physical regions of *Parth1.1*, *Parth2.1*, and *Parth3.1* (Fig. 5B). Three GWAS signals, *Par1.1*, *Par2.2*, and *Par3.5*, were located within the peak regions of QTLs *Parth1.1*, *Parth2.1*, and *Parth3.1* (Fig. 5C). LD decay (*r*) was analyzed to identify LD blocks around regions of peak QTNs, and multiple LD blocks also existed in each GWAS signal (Fig. 5D). Therefore, these three GWAS signals were selected for further investigation in this study.

**Fig. 6:**
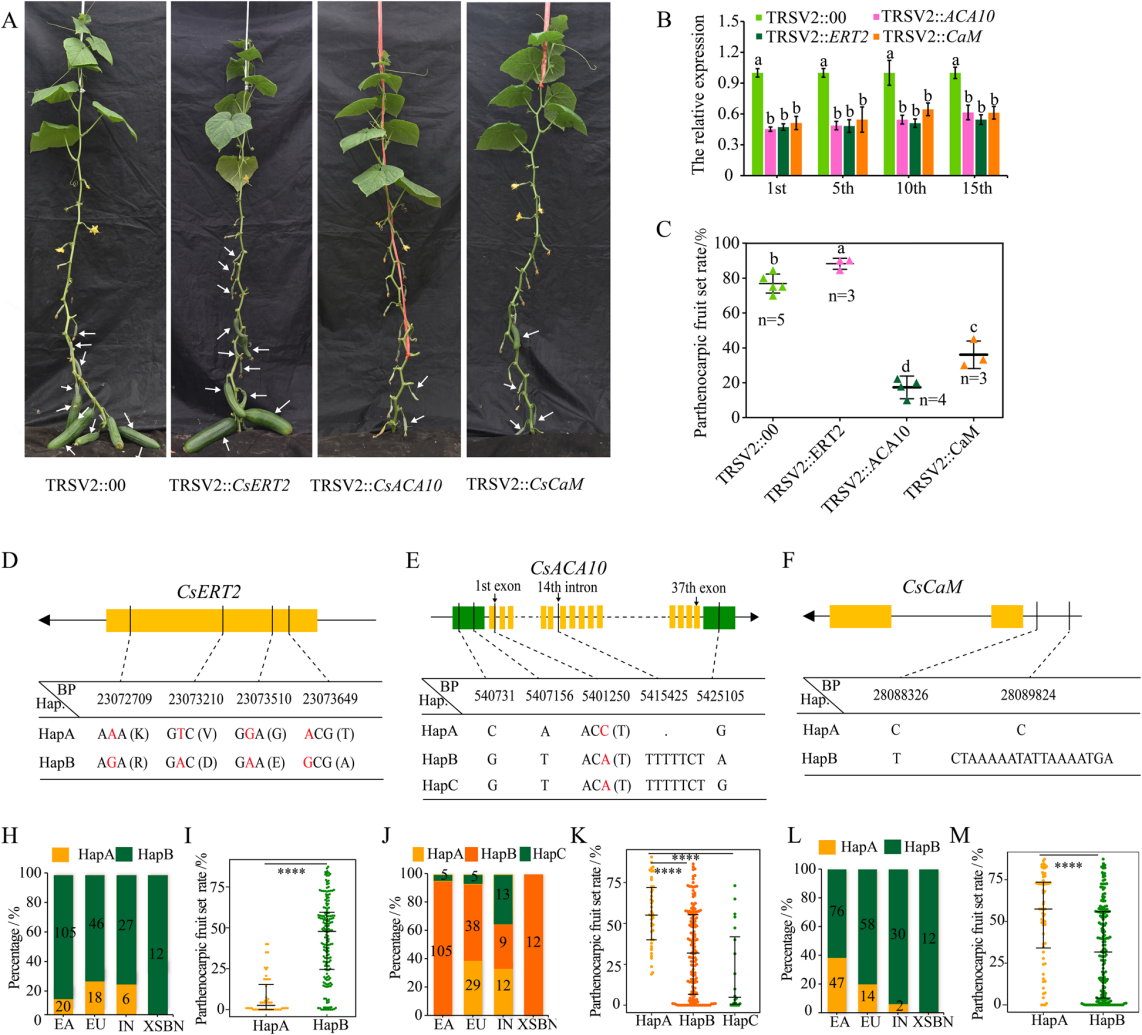
Validation of *CsERT2*, *CsACA10*, and *CsCaM* associated with parthenocarpy. **A** Phenotypic evaluation of parthenocarpy among wild-type (TRSV2::00), TRSV2::*CsERT2*, TRSV2::*CsACA10*, and TSRV2::*CsCaM* plants. The arrows represent parthenocarpic fruit, and the bar indicates 2 cm. **B** The relative expression levels of *CsERT2*, *CsACA10*, and *CsCaM* in VIGS lines were measured based on parthenocarpic fruits collected at 2 days on the 1st, 5th, 10th, and 15th fruit nodes. **C** Comparison of parthenocarpic fruit set rates between VIGS and wild-type plants. Important mutation sites located within the regions of *CsERT2* (**D**), *CsACA10* (**E**), and *CsCaM* (**F**). Haplotype frequencies of *CsERT2* (**H**), *CsACA10* (**J**), and *CsCaM* (**L**) in different cucumber groups. The parthenocarpic fruit set rates of different haplotypes of *CsERT2* (**I**), *CsACA10* (**K**), and *CsCaM* (**M**). ****p  < 0.0001, as determined by a two-tailed paired Wilcoxon test. The haplotype *CsERT2*^HapB^, *CsCaM*^HapA^, and *CsACA10*^HapA^ were PE11 genotype. The haplotype *CsERT2*^HapA^, *CsCaM*^HapB^, and *CsACA10*^HapB^ were PE12 genotype

### Functional validation of three parthenocarpic candidate genes associated with GWAS signals

The 50 kb regions of the GWAS signals *Par1.1*, *Par2.2*, and *Par3.5* around peak QTNs were further analyzed to identify candidate genes. By scanning the 9930 V3.0 genome, a total of 13 annotated candidate genes were identified in *Par1.2* (5), *Par2.2* (2), and *Par3.5* (6) (Fig. 5E). These candidate genes were selected for functional validation through a virus-induced gene silencing (VIGS) system. Gene expression and parthenocarpic ability were evaluated in VIGS lines and wild-type cucumber (TRSV2::00) from the 1st to 15th fruit nodes. We obtained at least three VIGS lines for each candidate gene, and the gene expression levels of parthenocarpic fruits were significantly lower than those of wild-type cucumber (Fig. S7). Compared with the wild-type cucumbers, only the VIGS lines of *CsaV3_1G037140* (*CsERT2*), *CsaV3_3G032560* (*CsCaM*), and *CsaV3_2G008960* (*CsACA10*) presented significant differences in parthenocarpic ability (Fig. 6A and 6C; Fig. S7E). The parthenocarpic fruit set rates of the *CsCaM* and *CsACA10* VIGS lines were lower than those of the wild-type PE11 lines by 30–45% and 70–86%, respectively (Fig. 6C). In contrast, the parthenocarpic fruit rates of the *CsERT2* VIGS lines were higher than those of the wild-type plants by 10–17% (Fig. 6C). These results indicate that *CsERT2, CsACA10,* and *CsCaM* are promising functional genes for the GWAS signals *Par1.1*, *Par2.2*, and *Par3.5*, respectively. *CsACA10* and *CsCaM* play positive roles associated with parthenocarpy, while *CsERT2* plays negative roles in parthenocarpy. *CsERT2* encodes an ethylene-responsive nuclear (ERT2)-like protein that is involved in ethylene signaling to regulate fruit development. *CsACA10* is a homologous gene of the *ACA* gene in *Arabidopsis* that encodes a calcium-transporting ATPase protein, which plays a crucial role in the transport of calcium ions within cells. *CsCaM* encodes a calcium-binding EF-hand family protein that serves as a key player in the calcium signaling pathway.

**Fig. 7:**
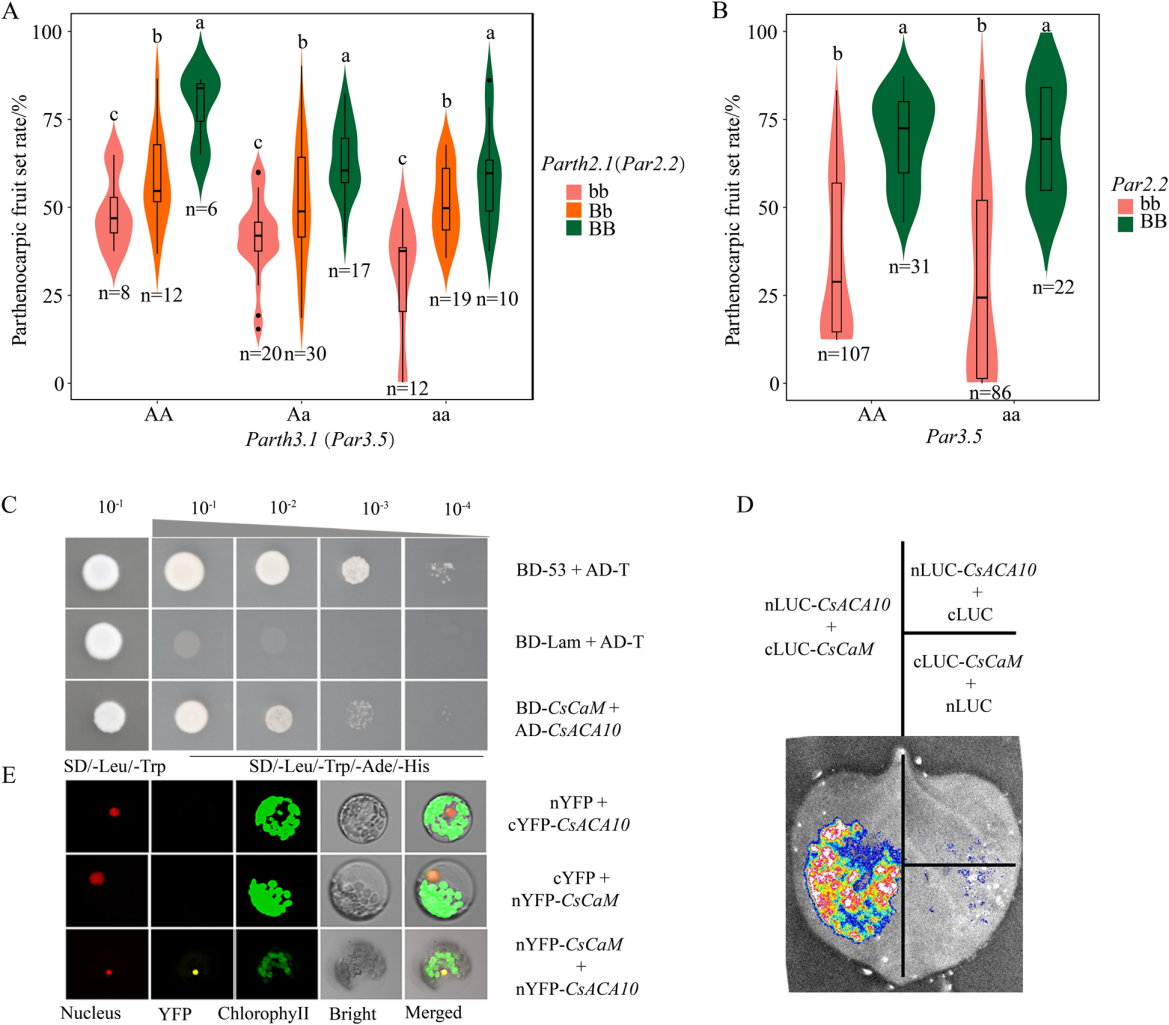
The synergistic effect of CsACA10 and CsCaM on enhancing parthenocarpy. Allelic combinations of *Par2.2* and *Par3.5,* and their effects on parthenocarpic ability in the mapping population (**A**) and 236 cucumber inbred lines (**B**). The *p* value between two groups was calculated via the Wilcoxon test. The alleles of *Parth2.1* and *Parth3.1* in the F_2:3_ mapping population are based on the peak markers SSR1353200 and UW085395, respectively. The alleles of *Par2.2* and *Par3.5* were determined on the basis of the peak SNPs in 236 cucumber inbred lines. AA (BB), Aa (Bb), and aa (bb) represent the heterozygous alleles of parthenocarpic lines, homozygous alleles of parthenocarpic lines, and heterozygous alleles of non-parthenocarpic lines, respectively. Interactions between CsACA10 and CsCaM were verified by a Y_2_H assay (**C**), LCA (**D**), and BiFC assay (**E**). The vectors pGBKT7-*p53* + pGADT7-T and pGBKT7-*Lam* + pGADT7-T were used as positive and negative controls, respectively, in the Y_2_H assay. The vectors nLUC-*CsACA10* + cLUC and cLUC-*CsCaM* + nLUC were used as negative controls in the LCA. The vectors nYFP + cYFP-*CsACA10* and nYFP-*CsCaM* + cYFP were used as negative controls in the BiFC assay. Each experiment was repeated independently three times with similar results

We also sequenced the alleles of 13 candidate genes across parent lines from the mapping population, including the region of the coding sequence and promoter, and identified *CsERT2, CsACA10,* and *CsCaM*, which harbored SNP or InDel mutations between parent lines (Fig. 6D, 6E, and F). Sequence alignment revealed four nonsynonymous SNPs in the exon of *CsERT2*, which resulted in changes in amino acids from Lys (K) to Arg (R), Val (V) to Asp (D), Gly (G) to Glu (E), and Thr (T) to Ala (A). Similarly, two SNPs, one SNP, one InDel, and one SNP were identified in the 5’UTR, first exon, 14th intron, and 3’UTR of *CsACA10*, respectively. One SNP and one InDel in the promoter region of *CsCaM* were identified, which might have caused differences in the gene expression levels. By combining the gene expression levels across parent lines and haplotype analysis, we found that cucumber inbred lines carrying the haplotype *CsERT2*^HapB^ presented lower gene expression levels and greater parthenocarpic ability than those with *CsERT2*^HapA^ (Fig. 6I, Fig. S8). The favorable haplotype *CsACA10*^HapA^ exhibited greater parthenocarpic ability compared with both *CsACA10*^HapB^ and *CsACA10*^HapC^ (Fig. 6K). Moreover, the haplotype *CsCaM*^HapA^ showed higher gene expression levels and greater parthenocarpic ability compared to *CsCaM*^HapB^ (Fig. 6M, Fig. S8). We also compared the haplotype frequency among cultivated cucumber groups and observed that the favorable haplotypes *CsERT2*^HapB^ and *CsCaM*^HapA^ were selected in the EA and EU cucumber groups (Fig. 6H and L), while the favorable haplotype *CsACA10*^HapA^ was selected in the EU cucumber group (Fig. 6J).

### CsACA10 exhibited synergistic effects with CsCaM on enhancing parthenocarpy

Notably, the QTL interaction between *Parth2.1* (*Par2.2*) and *Parth3.1* (*Par3.5*) explained 15% of the phenotypic variation (Figs. 7A and 7B; Table S13). The phenotypic data from the F_2:3_ population and 236 cucumber inbred lines revealed that *Par2.2* and *Par3.5* had synergistic effects on parthenocarpic ability, as the lines carrying both the *CsACA10* and *CsCaM* favorable alleles presented higher parthenocarpic fruit set rates than those with a single *CsACA10* favorable allele or *CsCaM* favorable allele (Figs. 7A and 7B). In addition, network analysis of protein‒protein interactions suggested that *CsPAT1* may interact with *CsCaM*. To determine the protein interaction between CsACA10 and CsCaM, a yeast two-hybrid (Y_2_H) assay, a luciferase complementation assay (LCA), and a bimolecular fluorescence complementation (BiFC) assay were performed for CsACA10 and CsCaM. As expected, the predicted interacting proteins were verified via the Y_2_H assay (Fig. 7C; Fig. S9 A) and LCA (Fig. 7D; Fig. S9B). Similarly, BiFC verified that nYFP-CsCaM interacted with cYFP-CsACA10 in the nucleus of *Arabidopsis thaliana* (Fig. 7E). These results revealed the synergistic effect of CsACA10 and CsCaM on enhancing parthenocarpic ability.

## Discussion

Many studies have highlighted the potential benefits of parthenocarpy in enhancing fruit yield and quality in plants. Currently, the genetic and molecular basis of parthenocarpy in Solanaceae crops has been extensively investigated. Compared with Solanaceae crops, parthenocarpy is more prevalent and complex in cucumber. In addition, the parthenocarpy trait has been selected in cucumber breeding for more than a hundred years, leading to significant changes in parthenocarpy among cultivated cucumbers. However, to date, the complex genetic and molecular basis of parthenocarpy in cucumber has largely not been elucidated.

### Modern cucumber breeding accelerates population divergence

Population structure, PCA, and phylogenetic tree analyses classified the 236 cucumber inbred lines into six cucumber groups (IN, EG, UP, NC, SC, and XSBN groups). This classification generally consistent with their geographical origins, likely because cucumbers from the same region exhibit relatively lower genetic diversity and thus tend to cluster together (Innark et al. 2013; Xu et al. 2024a, b). Comparative analysis with the four-group classification (IN, EU, EA, XSBN) proposed by Qi et al. (2013) revealed conserved clustering patterns: (i) the NC and SC groups clustered with the EA group, (ii) EG and UP groups clustered with EU groups, and (iii) IN and XSBN groups maintained their original classifications (Fig. S2C). Notably, we observed high structural similarity between UP and IN cucumber group, potentially attributed to significant genetic introgression between these groups. For example, many resistance genes, e.g., downy mildew resistance gene, were transferred from wild cucumber to UP cucumbers through hybridization in early cucumber breeding (Peterson et al. 1982; Wyszogrodzka et al. 1987; Wang et al. 2018). During modern breeding, gene exchange of parthenocarpy was occurred between the UP and EG groups (De Ponti et al. 1976; El-Shawaf & Baker 1981), and the SC and NC groups (Feng et al. 2020). These findings suggested that genetic exchange between different cucumber groups during modern breeding has accelerated divergence of cucumber group, leading to the formation of new cucumber groups.

### Multi-environment GWAS for determining the genetic basis of parthenocarpy in natural populations

The inheritance mode of parthenocarpy has been investigated in plants, ranging from single-gene inheritance to the inheritance of multiple quantitative trait loci. A total of nine QTLs for parthenocarpy, *pat*, *pat2*, *pat-k*, *pat3*, *pat4*, *pat4.1*, *pat4.2*, *pat5.1*, and *pat9.1*, have been identified in tomato (Mazzucato et al. 1998; Fos et al. 2000; Gorguet et al. 2004, 2008). Two major-effect QTLs associated with parthenocarpy were identified in eggplant (Miyatake et al. 2012). Unlike the parthenocarpy of Solanaceae crops, parthenocarpy is more complex and common in cucumber and is generally inherited in a dominant manner (Shnaider et al. 2018). Several genetic studies of parthenocarpy have focused on certain cucumbers and revealed at least 15 QTLs (Wu et al. 2016; Lietzow et al. 2016; He et al. 2019; Niu et al. 2020; Zhao et al. 2022; Gou et al, 2022; Devi et al. 2022).

To comprehensively assess the genetic basis of cucumber parthenocarpy in a natural population, we performed a GWAS for parthenocarpy based on 236 cucumber inbred lines across six environments. The ANOVA models of phenotypic variance indicated that genetic variation was the dominant source ( Table S4), which is consistent with the conclusions of Sun et al. (2006) and Wu et al. (2016). The broad-sense heritability value for parthenocarpy was high (*h*^*2*^ = 0.88), suggesting that these GWAS signals could be used effectively in marker-assisted breeding (MAS) of cucumber.

The GEMMA method detected seven parthenocarpy-associated GWAS signals (Figs. 3A; Table S6), which was significantly fewer than the number of QTLs identified through QTL mapping. This discrepancy may be functional limitations of GEMMA in detecting low-frequency and small-effect loci. In contrast, the IIIVmrMLM demonstrated precise identification of all genetic locus types, including small-effect genes, low-frequency alleles, and pleiotropic loci (Li et al. 2022a, b). Notably, IIIVmrMLM not only replicated six QTLs detected by GEMMA but also detected nearly all QTLs through QTL mapping (Figs. 3C; Table S6). Collectively, our analyses identified a total of 34 GWAS signals for parthenocarpy through GEMMA and IIIVmrMLM method (Fig. 3). Among them, three GWAS signals could be detected by an F_2:3_ mapping population, and 17 GWAS signals were colocalized with reported QTLs.

A number of promising candidate genes underlying GWAS signals have been confirmed or are suspected to be involved in the development of parthenocarpic fruit in cucumber or other crops, such as the hormone-related genes *CsARF10*, *CsERT2,* and *CsBES1*, the calcium signal-related genes *CsCaM* and *CsACA10* (Table S8). These results indicate the reliability of GWAS signals for parthenocarpy in this study. In total, the GWAS signals identified in this study were located within most reported QTL regions for parthenocarpy. Moreover, the sizes of the GWAS signals were smaller than those of the reported QTLs, providing a comprehensive summary of candidate genes for parthenocarpy. Recent work by Nie et al. (2025) reported *CsNPF1*, an AP2/ERF transcription factor, as a key regulator of parthenocarpic fruit development in cucumber. We observed that *CsNPF1* was not located GWAS signal regions identified in our study. While *CsNPF1* was detected in a sub-threshold peak region (*p* = 5.8 × 10^–5^, Fig. S10), it did not reach the genome-wide significance threshold (*p* = 1.5 × 10^–7^) established in this study. This discrepancy may be attributed to the genetic diversity of natural populations and the inherent limitations of GWAS detection power.

### Domestication of cucumber parthenocarpy through selection of different loci/genes

Seedless parthenocarpic fruits are not conducive to the propagation of offspring and are costly to the mother plant (Klap et al. 2017). Hence, the occurrence and permanence of the parthenocarpy trait in plants are largely affects of human domestication, with the goal of increasing yield and seedlessness (Varoquaux et al. 2000; Picarella et al. 2019). Recent studies revealed strong selection signals on *CsNPF1* during cucumber domestication (Nie et al. 2025). However, this gene was identified from a monogenic parthenocarpic mutant *npf1*, the natural cucumber populations exhibit polygenic regulation of parthenocarpy, suggesting that divergent domestication mechanisms in natural population. Notably, there is currently no direct evidence indicating that QTLs/genes associated with parthenocarpy in plants were selected during domestication.

In this study, our results revealed that 27 loci associated with parthenocarpy were subject to selection during the domestication of cucumber from a genomic perspective. The frequency of these favorable alleles in cultivated cucumbers was significantly greater than that in Indian cucumbers (Fig. S4), further supporting the positive selection of these parthenocarpic loci during domestication. We also found that only 8 parthenocarpy-related loci overlapped with these 112 domestication-selective sweeps from Qi et al. (2013), suggesting that these parthenocarpy-related QTLs have undergone selection in other natural populations (Table S10). The reasons for the inconsistency of different selected parthenocarpy-related loci may be as follows: on the one hand, we filtered out non-parthenocarpic cultivated cucumbers in the analysis to eliminate interference from other traits; on the other hand, the differences in cucumber samples of natural populations were used, as well as their resequencing depths. In addition, we observed that the number of selected parthenocarpic loci in XSBN cucumbers was significantly lower than that in Eurasian and East Asian cucumbers (Fig. 4G), possibly because of their long-term geographical isolation from other cucumber groups and retention of a few parthenocarpy-related loci from early domestication.

In the breeding of horticultural crops, hybrid breeding serves as an important driving force for enriching favorable trait genes. In this study, the favorable alleles for parthenocarpy were distributed among different IN cucumbers, while favorable alleles were enriched in cultivated cucumbers with high parthenocarpic ability, which may have resulted from the enrichment of favorable alleles by hybrid breeding during the early domestication of cucumbers (Feng et al. 2020). Furthermore, hybrid breeding among different cucumber groups may have accelerated the domestication of parthenocarpy in cucumbers. For example, favorable alleles associated with parthenocarpy were transferred from European greenhouse cucumbers to American processing cucumbers through hybrid breeding (De Ponti et al. 1976). In addition, some selective sweeps of this study overlapped with genes/QTLs of other domestication-related traits, including fruit size, bitterness, and gynoecy (Table S9), indicating that these traits might have undergone a second round of selection during the domestication of parthenocarpy. We also noted that a significant proportion of the sweep selection signals did not overlap with any cloned domestication genes/QTLs. These sweep selection signals could represent geographical genetic signatures or reflect unidentified selection pressures of cultivation practices (Huang et al. 2022).

### Roles of the ethylene and calcium signaling pathways in the regulation of cucumber parthenocarpy

Parthenocarpy is closely related to plant hormones. Many hormone-related genes have been shown to contribute to the initiation of parthenocarpic fruit growth in tomato. For example, the downregulation of the auxin response factors *SlARF5* (Liu et al. 2018), *SlARF8* (Goetz et al. 2006), *SlIAA9* (Wang et al. 2005), and *SlARF7* (De Jong et al. 2011) results in the formation of parthenocarpic fruit. In cucumber, some evidence suggests that genes related to hormones are involved in the development of parthenocarpic fruit. For example, the exogenous application of plant growth hormones, including auxins, cytokinins, gibberellic acids, brassinosteroids, and ethylene biosynthesis inhibitors (Ogawa et al. 1989; Fu et al. 2008, 2010; Hikosaka et al, 2015; Li et al, 2017; Meng et al, 2024), has been shown to enhance or regulate parthenocarpic fruit growth in cucumber. While several auxin-related genes of cucumber have been confirmed to regulate parthenocarpy (Xu et al. 2017), the regulatory mechanisms of parthenocarpy mediated by other hormone-related genes have not yet been clarified. In this study, the role of a novel ethylene nuclear perception gene, *CsERT2*, in the regulation of parthenocarpy in cucumber was validated. The downregulation of *CsERT2* transcription was found to increase parthenocarpic ability (Fig. 6), which is consistent with previous studies showing that ethylene negatively regulates parthenocarpy in cucumber (Martínez et al. 2013; Shinozaki et al. 2015).

The calcium signaling pathway is also involved in the regulation of parthenocarpic fruit development in tomato. *SlSUN* encodes an IQ domain protein, and the overexpression of *SlSUN* in tomato induces parthenocarpic fruit growth (Wu et al. 2011). Similarly, *CsCaM* from this study is a homologous gene of *SlSUN*, and the downregulation of *CsCaM* expression reduced parthenocarpic ability in cucumber (Fig. 6). *CsACA10* is additionally linked to the calcium signaling pathway and encodes a calcium pump protein. Suppressing the expression of *CsACA10* also significantly decreased parthenocarpic ability in cucumber (Fig. 6). Several studies have suggested that the IQD family facilitates and specifically binds CaM/CML to activate or enhance the function of the ACA gene family in transporting calcium ions in *Arabidopsis* (Hwang et al. 2000; Bürstenbinder et al. 2017). Correspondingly, this study validated the protein interaction between CsCaM and CsACA10. Although there is no evidence in our study that the interaction between CsCaM and CsACA10 enhances the enzymatic activity of CsACA10, the phenotypic data revealed that the interaction between CsCaM and CsACA10 can improve the parthenocarpy of cucumber. Therefore, we speculate that calcium signaling plays a positive role in enhancing parthenocarpic ability in cucumbers. These results validate the contributions of ethylene and calcium signals in regulating parthenocarpy in cucumber. Nonetheless, further work is needed to elucidate the mechanisms by which the CsERT2-mediated ethylene signaling network and the CsACA10/CsCaM-mediated calcium signaling network contribute to parthenocarpy through the verification of transgenic approaches, transcriptomics, proteomics, and other multi-omics strategies.

## Methods

### Plant material and growth conditions

The mapping association panel consisted of 236 cucumber inbred lines that had been self-pollinated over 6 generations at Nanjing Agricultural University, Nanjing, China. These cucumber inbred lines were collected from 24 provinces of China, as well as from India, the USA, the Netherlands, Germany, France, Israel, Turkey, South Korea, and Japan, covering the main cucumber cultivation area (Table S1; Fig. S1). The 123 F_2:3_ population was constructed for QTL mapping, with parent lines (PE11 and PE12) selected from the association panel. The 236 cucumber inbred lines were grown under greenhouse conditions in six environment trials (three years × two seasons), including the spring of 2018 (env_1), the fall of 2018 (env_2), the spring of 2021 (env_3), the fall of 2021 (env_4), the spring of 2022 (env_5), and the fall of 2022 (env_6) at the Baima experimental base (119.02 N, 31.65 E) of Nanjing Agricultural University, Nanjing, China. The F_2:3_ population was grown under greenhouse conditions in the spring of 2023 (env_1) and the fall of 2023 (env_2) at the Baima experimental base. All the experiments were conducted with a randomized complete block design (RCBD). Six individual plants from the 236 cucumber inbred lines were replicated two times, and ten individual plants from the 123 F_2:3_ population were replicated two times. Individual plants were spaced 30 cm apart in rows, and the rows were 80 cm apart.

### Evaluation of parthenocarpy

Individual plants from the 236 cucumber lines and the 123 F_2:3_ population were used for measurements of parthenocarpic ability. In this study, we focused on the total parthenocarpic ability of the main stem from 5 to 40th fruit node, and all lateral branches were removed. All female flowers of each plant were trapped with colorful metal wire to prevent pollen contamination before anthesis (Supplemental Fig. 1). Due to the difference of sex type of each cucumber inbred lines, the number of trapped ovaries for individual cucumber in each environment ranged from 6 to 25. The trapped ovaries that displayed any signs of growth and expansion at 10 days after anthesis were counted as parthenocarpic fruits, including normal parthenocarpic fruits, malformed parthenocarpic fruits, and parthenocarpic dormant fruits, where the trapped ovaries sometimes initiated growth but ceased at any point during development. The parthenocarpic fruit set rate is widely used to assess the inconsistent fruit number of cucumber populations (Sun et al. 2006; Wu et al.; 2016). The parthenocarpic ability of each plant was measured by $\text{PFS rate}=\frac{\text{Number of parthenocarpic fruits }}{\text{Total trapped ovaries}}$ according to general methods of Wu et al. (2016). To minimize nutritional competition among fruits, all developed parthenocarpic fruits were removed after phenotypic evaluation. For each replication, the mean parthenocarpic fruit set rate of all the plants was calculated to assess the parthenocarpic ability of the cucumber inbred lines or family lines.

### Phenotypic data analysis

A geographical map of the 236 cucumber lines was constructed via the ‘ggmap’ package in R. The package ‘PerformanceAnalytics’ was used to estimate correlation analysis between different environments in R. The significance of the mean values was calculated between two groups via the Wilcoxon test in R. The best linear unbiased prediction (BLUP) for parthenocarpy across the six environments was calculated using the package ‘lme4’ in R via the mixed linear model: BLUP = ((1|G) + (1|S) + (1|R) + (1|G:S) + (1|G:R) + (1|R:S)), with genotype (G), season (S), replication (R), genotype × season (G:S), genotype × replication (G:R), and replication × season (R:S) as random effects. The broad-sense heritability (*h*^2^) was estimated via the following formula: *h*^2^ $=\frac{{{\upsigma }^{2}}_{\text{G}}}{{{\upsigma }^{2}}_{\text{G}}+\frac{{{\upsigma }^{2}}_{\text{G}\times \text{R}}}{\text{r}}+\frac{{{\upsigma }^{2}}_{\text{G}\times \text{S}}}{\text{s}}+\frac{{{\upsigma }^{2}}_{\upvarepsilon }}{\text{rs}}}$, where σ^2^_G_ represents the genotypic variance, and σ^2^_G×R_ and σ^2^_G×S_ represent the interaction variances between genotype and replication and between genotype and season, respectively. σ^2^_ε_ represents the residual variance. r and s are the number of replications and the number of seasons, respectively. The significant difference analysis of different group was calculated the Wilcoxon test in R.

### DNA extraction and sequencing

The genomic DNA was extracted from young leaves via a Plant Genomic DNA Extraction Kit **(**Vazyme, China**)**. At least 1 μg of genomic DNA from each cucumber line was used to construct a sequencing library according to the instructions of Illumina. Paired-end libraries with an insert size of ~ 450 bp were prepared and sequenced by Shanghai Biozeron Biotechnology Co., Ltd. (Shanghai, China) using the Illumina HiSeq PE 2 × 150 bp read length. These raw paired-end reads from this study were trimmed and quality controlled according to Trimmomatic with default parameters. The high-quality sequencing reads were aligned to the cucumber reference genome of 9930 V3.0 using BWA software in “bwa mem” mode (http://biobwa.sourceforge.net/). SNP calling was performed using the Haplotype Caller function from GATK (http://www.broadinstitute.org/gatk/). The SNP dataset was filtered by removing SNP variations with QD < 2.0, FS > 60.0, MQ < 40.0, and SOR > 10.0.

### Phylogenetic and population structure analyses

After filtering the SNP dataset to retain SNPs with ≤ 50% missing data and a minor allele frequency (MAF) > 0.05, we obtained a total of 1,283,903 SNPs for phylogenetic and population structure analysis. A neighbor‒joining tree was constructed, and the results were visualized via MEGAX. PCA was performed through GCTA software with default settings (Yang et al. 2011). The population structure of the 236 cucumber inbred lines was inferred via fastSTRUCTURE, and the input parameter population clusters (K) were set from 2 to 10 (Raj et al. 2014). The pattern of LD decay for the six cucumber groups was calculated via PopLDdecay (Zhang et al. 2019). The F_ST_ between different cucumber groups and π were calculated via VCFtools with a window size of 50 kb and a step size of 100 bp. Multidimensional scaling was performed to visualize the F_ST_ values among different cucumber groups using the ‘cmdscale’ function in R (Table S9).

### GWAS for parthenocarpy

The GEMMA method has been widely adopted for detecting QTNs in complex trait analyses, as it effectively accounts for population structure and confounding factors (Zhou & Stephens 2012). However, to address limitations in effect estimation accuracy and polygenic background control, Li et al. (2022a, b) developed the IIIVmrMLM model, which is a novel GWAS framework capable of identifying all genetic loci types with near-unbiased effect estimates, high statistical power, and reduced false-positive rates. Base on the complementary strengths between GEMMA and 3IIIVmrMLM method, we employed both methods to systematically investigate the genetic basis underlying cucumber parthenocarpy. We filtered the SNP dataset to include only those with ≥ 20% missing data and a minor allele frequency (MAF) < 0.05. We subsequently identified a total of 1,387,845 SNPs (Fig. S3) for GWAS using the LMM model of GEMMA software and the IIIVmrMLM QEI package of R scripts. The K and Q matrix of the population structure was selected as a random effect in the GWAS. The plots of Manhattan and quantile‒quantile (Q‒Q) were produced using the ‘CMplot’ package. The suggestive thresholds (0.05/number of SNPs) were set at *p* = 3.60 × 10^–8^ on the basis of Bonferroni correction. However, the observed *p* value of many potentially significant loci was greater than the suggestive *p* value; hence, the thresholds were set at *p* = 1.5 × 10^–7^ to identify significant GWAS signals for GEMMA. The suggestive *p* value for IIIVmrMLM was set at 1.0 × 10^–3^ with the default setting (Li et al. 2022b). To obtain independent GWAS signals, multiple SNPs exceeding the threshold in a 500 kb region were considered the same GWAS signals.

### Selective sweep detection for parthenocarpy during cucumber domestication

Within IN cucumber group, we identified one cucumber was wild cucumber *C. sativus* var. *hardwickii*, and 9 *C. sativus* var. *hardwickii* variants that exhibit characteristic wild traits, including small bitter fruits, diminutive leaves, and absence of parthenocarpic ability. The remaining 24 cucumbers were classified as Indian cultivated cucumbers, also showed low parthenocarpy and their parthenocarpy has not been subjected to human selection. Hence, 34 IN cucumbers, as the wild cucumber group, and 134 cultivated cucumbers, as the domestication cucumber group, were selected to identify domestication-related sweeps through genome-wide domestication analyses. The parthenocarpic ability of the cultivated cucumber group was greater than 10% to eliminate interference from other traits. To identify the selectively related sweep regions between different cucumber groups and the IN group, the 96 EA, 76 EU, and 12 XSBN cucumbers were compared with the 34 IN cucumbers. The XP-CLR and F_ST_ methods, with a step size of 50 kb and a sliding window of 100 bp, were used to identify genome-wide selective sweeps for parthenocarpy. The top 5% value of Fst or XP-CLR were calculated by function sort and head 5% lines in bash. The common top 5% of the F_ST_ and XP-CLR regions were identified as putatively selective sweeps using intersect function of BEDTools package.

### QTL analysis

The F_2:3_ populations of PE11 × PE12 were used to detect QTLs for parthenocarpy. A genetic linkage map was constructed using 161 polymorphic markers, including 116 SSR and 45 InDel markers. The genetic map covered 839.49 cM for seven chromosomes, with an average marker interval of 5.21 cM (Table S12). We performed QTL analysis through the composite interval mapping function of the ‘r/qtl’ package. The QTL threshold was calculated on the basis of 1000 permutations, and the intervals of significant QTLs were determined using a 1.5 logarithm of odds (LOD) interval. The interaction between two QTLs was calculated by ‘fitqtl’ function in r/qtl with model formula: y ~ Q1 + Q2 + Q3 + Q1:Q2.

### Virus-induced gene silencing system

A tobacco ringspot virus-based vector system (Fang et al. 2021) containing two plasmids, pTRSV1 and pTRSV2, was used for gene silencing of candidate genes. The specific coding sequences of the 13 candidate genes were designed according to the design website of the gene silencing sequence (https://vigs.solgenomics.net/). The 13 specific coding sequences were amplified from the genomic sequence of PE11 and cloned and inserted into the vector of tobacco rattle virus (pTRSV2) via a Hieff clone plus one step cloning kit (Yeasen, China). The VIGS vectors were subsequently transferred into the *Agrobacterium tumefaciens* strain GV3101. The *Agrobacterium tumefaciens* strain mixture containing the pTRSV2-specific coding sequence and pTRSV1 was agroinfiltrated into the cotyledons of the parthenocarpic line PE11. The cotyledons of cucumber were cultivated in darkness for three days at 25 °C. The gene silencing efficiency was confirmed by qRT-PCR with primers specific to target genes. VIGS lines were grown in a plant growth chamber under temperature conditions (18 °C/15 °C, day/night) for three weeks before transplanting to field conditions at the Baima experimental station. To systematically evaluate the phenotypic consequences of gene silencing, parthenocarpic fruits from the 1 st, 5th, 10th, and 15th fruit nodes were collected to assess the expression levels of the target genes. The primers used are listed in Table S14.

### qRT–PCR analysis

Parthenocarpic fruit or ovary samples from parent lines of the mapping population and VIGS lines were collected for qRT–PCR. Total RNA was isolated via an RNA extraction kit (TransZol reagent; TransGene) according to the manufacturer’s instructions. qRT–PCR was carried out in a total volume of 10 µl using TB Green Premix Ex Taq II (Takara, China) in a Bio-Rad CFX96 Real-Time System (Bio-Rad, Hercules, CA, USA). The expression levels of the candidate genes were calculated via the relative quantification method (Livak and Schmittgen 2001). The primers used are listed in Table S14.

### Yeast two-hybrid (Y_2_H) assay

For the Y_2_H assay, pGADT7 and pGBKT7 were used to identify protein interactions. The full-length coding sequences of *CsACA10* and Cs*CaM* were fused with pGBKT7 and pGADT7 via the Hieff clone plus one step cloning kit (Yeasen, China). Yeast transformation was performed following the manufacturer’s instructions. The primers used are listed in Table S14.

### Bimolecular fluorescence complementation (BiFC) assay

The full-length coding sequences of *CsACA10* (without stop codons) and *CsCaM* (without stop codons) were cloned and inserted into the pSPYCE (cYFP) vector and pSPYNE (nYFP) vector, respectively. The extraction of *Arabidopsis thaliana* protoplasts was performed using a PPT101 extraction kit (Coolaber, Beijing) according to the manufacturer’s instructions. All the plasmids for the BiFC assay were subsequently transformed into *Arabidopsis thaliana* protoplasts via the PEG-mediated method. After 18 h of coinfiltration, YFP signals from the protoplasts were observed using a confocal laser scanning microscope (Zeiss LSM780). The primers used are listed in Table S14.

### Luciferase complementation (LCA) assay

The full-length coding sequences of *CsACA10* and *CsCaM* were amplified and cloned and inserted into the nLUC and cLUC plasmid vectors, respectively. The two vectors were subsequently transferred into the *A. tumefaciens* strain GV3101. *Agrobacterium tumefaciens*-mediated transient expression was conducted in one-month-old tobacco. D-luciferin potassium salt (1 mM, YEASEN, China) was added to the tobacco leaves, and the samples were incubated in the dark for 10 min. Then, a low-light cooled CCD imaging apparatus (PIXIS 1024B, USA) was used to obtain the fluorescent signal of LUC. Three independent experiments were performed for the luciferase complementation assay (LCA). The primers used for the LCA are listed in Table S14.

## Supplementary Information

Supplementary Material 1. Fig. S1 236 cucumber inbred lines and evaluation of parthenocarpy in these cucumber lines. A Fruit morphology of the 236 cucumber inbred lines. Parthenocarpic fruit growth includes parthenocarpic fruit expansion (B) that the unpollinated ovaries develop into commercial, and fruits and parthenocarpic dormant fruit(C) that the trapped ovaries activated growth but ceased at any point during development. Aborted parthenocarpic fruit (D, E) that unpollinated ovaries did not exhibit fruit development and gradually became inactive withered. The white bars represent 10 cm, 2 cm, 2 cm, and 2 cm in A, B, C, and D, respectively. Fig. S2 Analysis of the population structure among 236 cucumber lines. A The population structure based on the model of admixture at K = 6 represented the best model for diverging the six distinct groups of IN, XSBN, UP, EG, SC, and NC. B The population structure was analyzed via PCA using the first two principal components. C The population structure of 351 cucumber lines, among which the resequencing data of 115 cucumber lines were obtained from Qi et al. (2013). As expected, the phylogenetic structure of 236 cucumber lines was consistent with the phylogenetic structure of 115 cucumber lines, with four classic cucumber groups, IN, XSBN, EA (SC, NC), and EU (UP, EG). D Summary of nucleotide diversity for the six cucumber groups using violin plots. The different letters indicate significant differences at the *p* < 0.05 level as determined by the Wilcoxon test. Fig. S3 Distribution of 1,306,756 high-quality SNPs for GWASs on the cucumber chromosome. The color indicates the density of the SNP distribution; gray areas have no markers, while greener markers indicate lower density and redder markers indicate higher density. Fig. S4 Haplotype frequency of 27 GWAS signals in four groups among 236 GWAS populations. For each GWAS signal, Hap.H represents cucumbers with a high parthenocarpic fruit set rate, whereas Hap.L represents those with a low parthenocarpic fruit set rate. **, ***, and **** denote significant differences at *p* < 0.01, *p* < 0.001, and *p* < 0.0001, respectively, as determined by the Wilcoxon test. Fig. S5 Phenotypic data of the parental lines, their F_1_ (A), and the mapping population F_2:3_ across the two environments (B). **** indicates *p* < 0.0001, as determined via the Wilcoxon test. Fig. S6 QTL mapping for parthenocarpy using an F_2:3_ population derived from a cross between PE11 and PE12. Three QTLs associated with parthenocarpy were detected on the chromosome of cucumber in two environments (A): *Parth1.1* (B), *Parth2.1* (C), and *Parth3.1* (D). Fig. S7 Validation of the 13 candidate genes associated with parthenocarpy via virus-induced gene silencing. The relative expression of 13 candidate genes in their VIGS lines was detected on the basis of parthenocarpic fruits collected at 2 days on the 1st (A), 5th (B), 10th (C), and 15th fruit nodes (D). E Comparison of the parthenocarpic fruit set rates of VIGS and wild-type plants. There were significant differences in parthenocarpic ability between the *CsaV3_1G037140*, *CsaV3_2G008960,* and *CsaV3_3G032560* VIGS lines and the wild-type cucumber line TRSV2::00. The data in A, B, C, D, and E are the means ± SDs, and significant differences at the *p* < 0.05 level, as determined by the Wilcoxon test, are indicated by different letters. Fig. S8 Relative expression of 13 candidate GWAS genes from parthenocarpic fruits at -2 days, 0 days, and 2 days. **** indicates *p* < 0.0001, as determined by the Wilcoxon test. Fig. S9 Autoactivation verification assay of BD-CsCaM (A) and LUC enzyme activity assay (B). pCL1 and BD served as positive and negative controls in autoactivation verification assay, respectively; nLUC-CsACA10+cLUC and cLUC-CsCaM+nLUC were used as negative controls. The significant differences at the *p* < 0.05 level, as determined by the Wilcoxon test, are indicated by different letters. Fig. S10 GWAS signals associated with parthenocarpy near CsNPF1. The blue line indicates significance threshold (*p* =1.5×10^-7^) of GEMMA. The red arrow indicates *p* value of *CsNPF1*.

Supplementary Material 2. Table S1: Summary of the 236 cucumber inbred lines sampled. Table S2: Structure analysis of 236 cucumber lines via PCA and fastSTRUCTURE (v1.0). Table S3: Pairwise FST values between different cucumber groups. Table S4: The means, minimums, maximums, variances, coefficients of variation (CVs) of parthenocarpic ability in the six cucumber groups, ANOVA results, and broad-sense heritability (*h*^*2*^) values of all inbred lines. Table S5: QTLs detected for parthenocarpy in cucumber on the basis of a previous study. Table S6: Summary of GWAS signals associated with parthenocarpy identified in this study. Table S7: List of candidate genes within 25 kb upstream and downstream of the peak QTNs of GWAS signals. Table S8: List of candidate parthenocarpy-related genes for cucumber based on known parthenocarpy-related genes in plants. Table S9: Putative sweep regions during cucumber domestication for parthenocarpy and a list of related domestication genes within the putative sweep regions. Table S10: The parthenocarpic loci of this study overlapped with the domestication sweep regions from Qi (Qi et al. 2013) on the basis of the 9930 V2.0 physical region. Table S11: Summary of favorable alleles identified from GWAS and genome-wide domestication analyses. Table S12: Information on the markers on the genetic map of PE11 × PE12. Table S13: QTLs for parthenocarpy detected with composite interval mapping (CIM) in the present study. Table S14: List of primer sequences used in this study.

## Data Availability

We confirm that the data supporting the results are available in the present article and its Supplementary information.

## Abbreviations

BiFC: Bimolecular fluorescence complementation
BLUP: Best linear unbiased prediction
EG: European greenhouse
F_ST_: Fixation index
GWAS: Genome-wide association study
IN: India
InDel: Insertions and deletions
LCA: Luciferase complementation assay
LD: Linkage disequilibrium
LUC: Firefly luciferase
NC: North China
PCA: Principal component analysis
PFS: Parthenocarpic fruit set
PVE: Phenotypic variance explained
qRT-PCR: Quantitative real-time PCR
QTL: Quantitative trait loci
QTNs: Quantitative trait nucleotides
SC: South China
SNP: Single-nucleotide polymorphisms
UP: U.S processed
VIGS: Virus-induced gene silencing
XP-CLR: Cross-population composite likelihood ratio
Y_2_H: Yeast two-hybrid
