# Supplementary material for: Integrated genome-wide domestication and association analyses reveal the complex genetic basis of parthenocarpy during cucumber domestication
Source: Mol Hortic. 2026 Jan 7;6:7. doi: 10.1186/s43897-025-00182-y (PMC12777003; doi:10.1186/s43897-025-00182-y)
Supplement: Supplementary file 1 — Supplementary Material 1. Fig. S1 236 cucumber inbred lines and evaluation of parthenocarpy in these cucumber lines. A Fruit morphology of the 236 cucumber inbred lines. Parthenocarpic fruit growth includes parthenocarpic fruit expansion (B) that the unpollinated ovaries develop into commercial, and fruits and parthenocarpic dormant fruit(C) that the trapped ovaries activated growth but ceased at any point during development. Aborted parthenocarpic fruit (D, E) that unpollinated ovaries did not exhibit fruit development and gradually became inactive withered. The white bars represent 10 cm, 2 cm, 2 cm, and 2 cm in A, B, C, and D, respectively. Fig. S2 Analysis of the population structure among 236 cucumber lines. A The population structure based on the model of admixture at K = 6 represented the best model for diverging the six distinct groups of IN, XSBN, UP, EG, SC, and NC. B The population structure was analyzed via PCA using the first two principal components. C The population structure of 351 cucumber lines, among which the resequencing data of 115 cucumber lines were obtained from Qi et al. (2013). As expected, the phylogenetic structure of 236 cucumber lines was consistent with the phylogenetic structure of 115 cucumber lines, with four classic cucumber groups, IN, XSBN, EA (SC, NC), and EU (UP, EG). D Summary of nucleotide diversity for the six cucumber groups using violin plots. The different letters indicate significant differences at the p < 0.05 level as determined by the Wilcoxon test. Fig. S3 Distribution of 1,306,756 high-quality SNPs for GWASs on the cucumber chromosome. The color indicates the density of the SNP distribution; gray areas have no markers, while greener markers indicate lower density and redder markers indicate higher density. Fig. S4 Haplotype frequency of 27 GWAS signals in four groups among 236 GWAS populations. For each GWAS signal, Hap.H represents cucumbers with a high parthenocarpic fruit set rate, whereas Hap.L represents those [file 43897_2025_182_MOESM1_ESM.pdf]

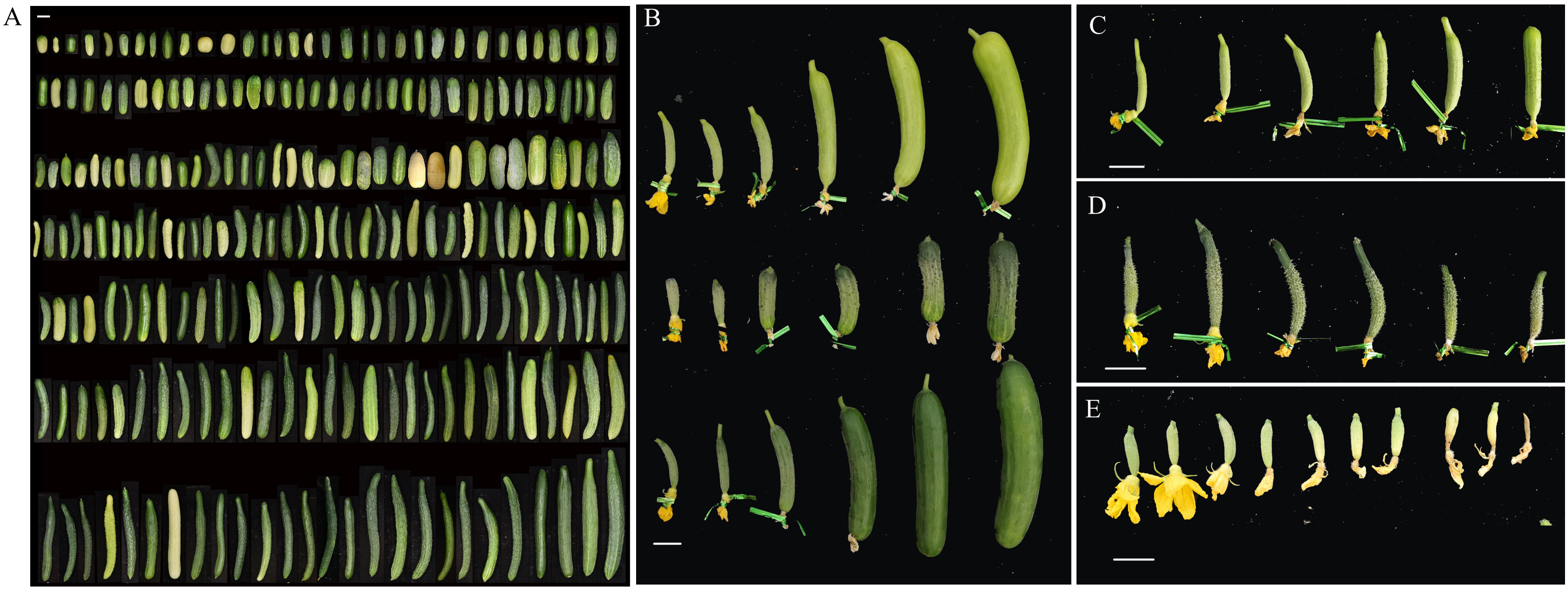

Fig. S1 236 cucumber inbred lines and evaluation of parthenocarpy in these cucumber lines. **A** Fruit morphology of the 236 cucumber inbred lines. Parthenocarpic fruit growth includes parthenocarpic fruit expansion (**B**) that the unpollinated ovaries develop into commercial fruits, and parthenocarpic dormant fruit (**C**) that the trapped ovaries activated growth but ceased at any point during development. Aborted parthenocarpic fruit (**D**, **E**) that unpollinated ovaries did not exhibit fruit development and gradually becameinactive withered. The white bars represent 10 cm, 2 cm, 2 cm, and 2 cm in **A**, **B**, **C**, and **D**, respectively.

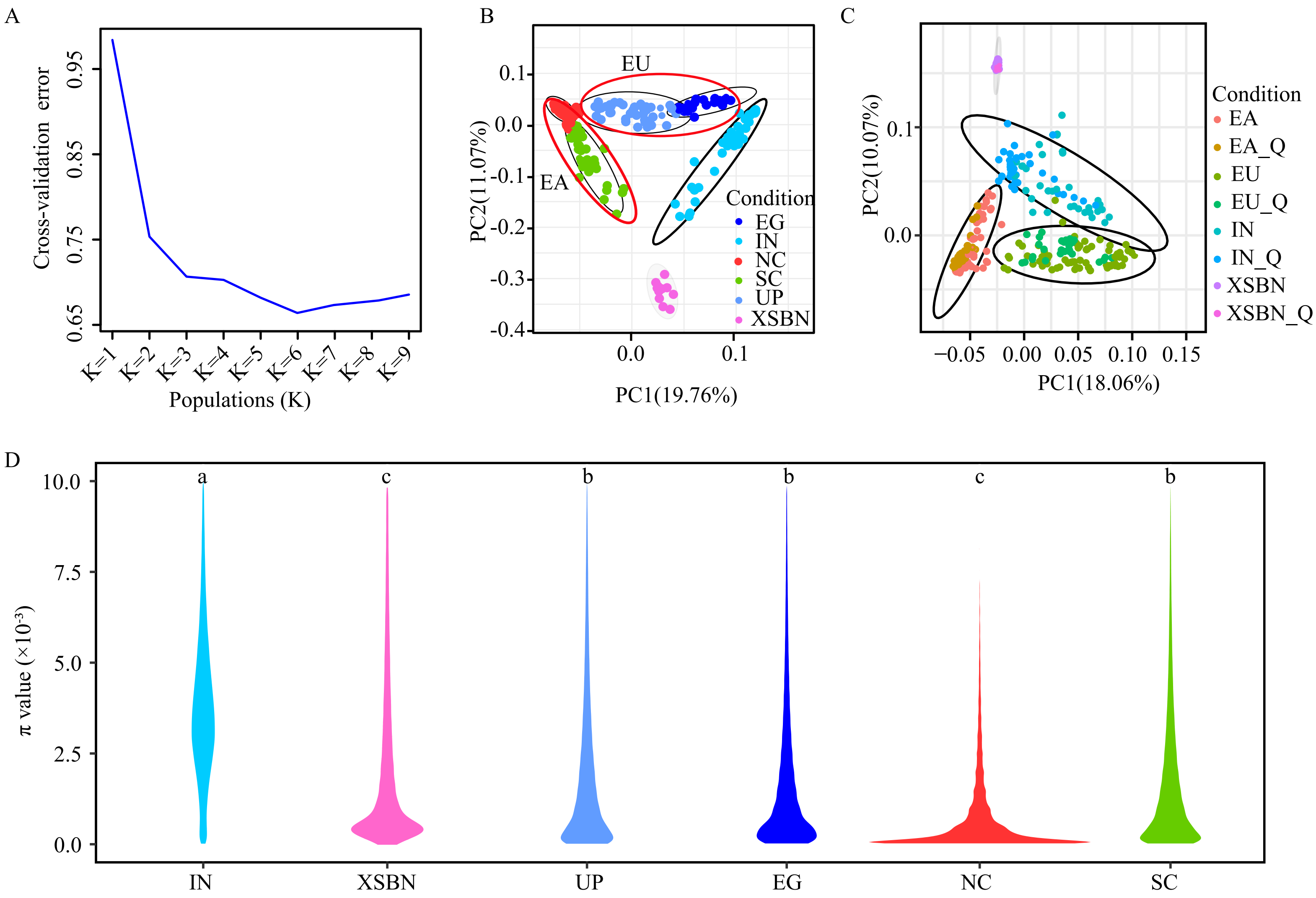

Fig. S2 Analysis of the population structure among 236 cucumber lines. **A** The population structure based on the model of admixture at K = 6 represented the best model for diverging the six distinct groups of IN, XSBN, UP, EG, SC, and NC. **B** The population structure was analyzed via PCA using the first two principal components. **C** The population structure of 351 cucumber lines, among which the resequencing data of 115 cucumber lines were obtained from Qi et al. (2013). As expected, the phylogenetic structure of 236 cucumber lines was consistent with the phylogenetic structure of 115 cucumber lines, with four classic cucumber groups, IN, XSBN, EA (SC, NC), and EU (UP, EG). **D** Summary of nucleotide diversity for the six cucumber groups using violin plots. The different letters indicate significant differences at the $p < 0.05$ level as determined by the Wilcoxon test.

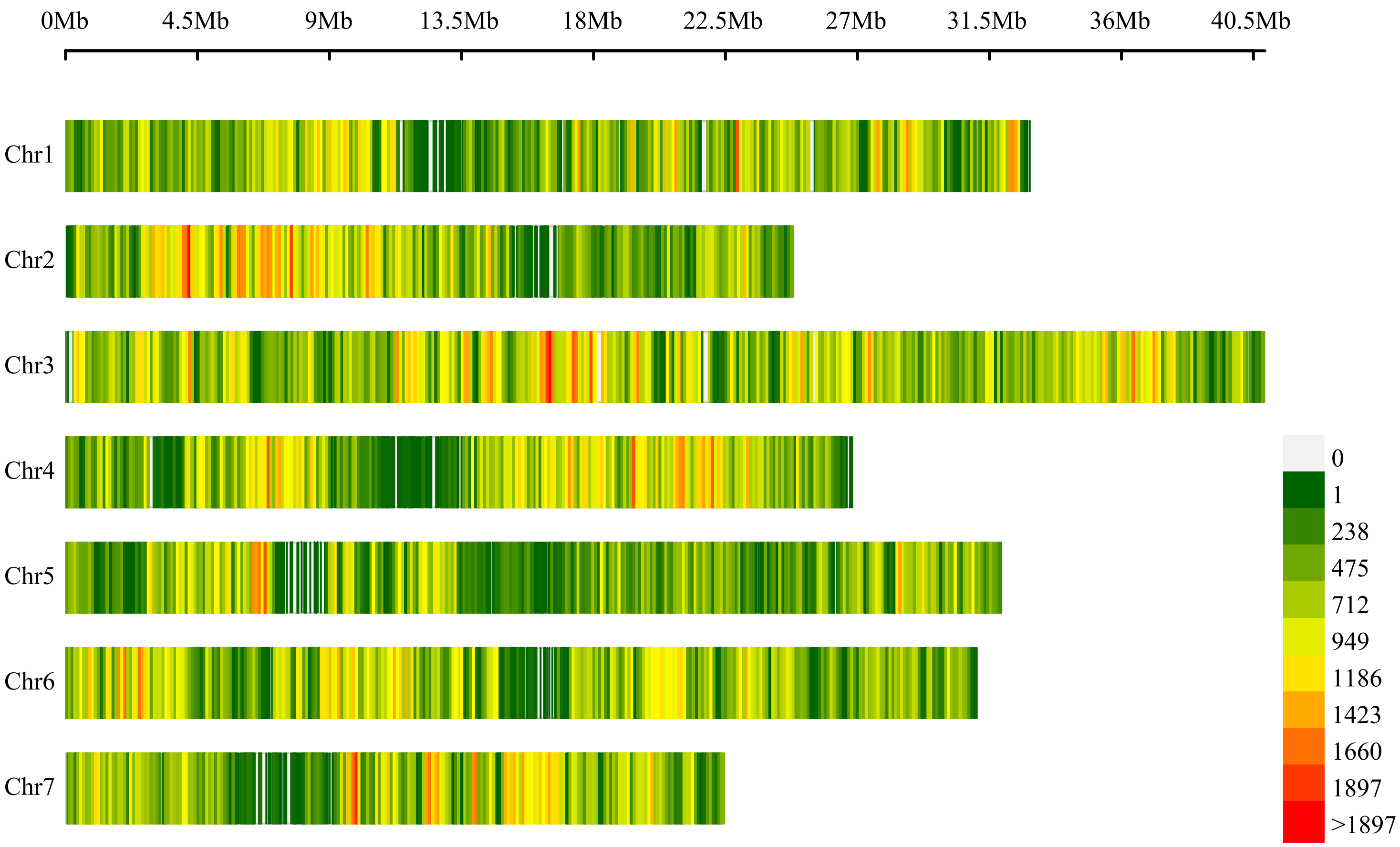

Fig. S3 Distribution of 1,306,756 high-quality SNPs for GWASs on the cucumber chromosome. The color indicates the density of the SNP distribution; gray areas have no markers, while greener markers indicatelower density and redder markers indicate higher density.

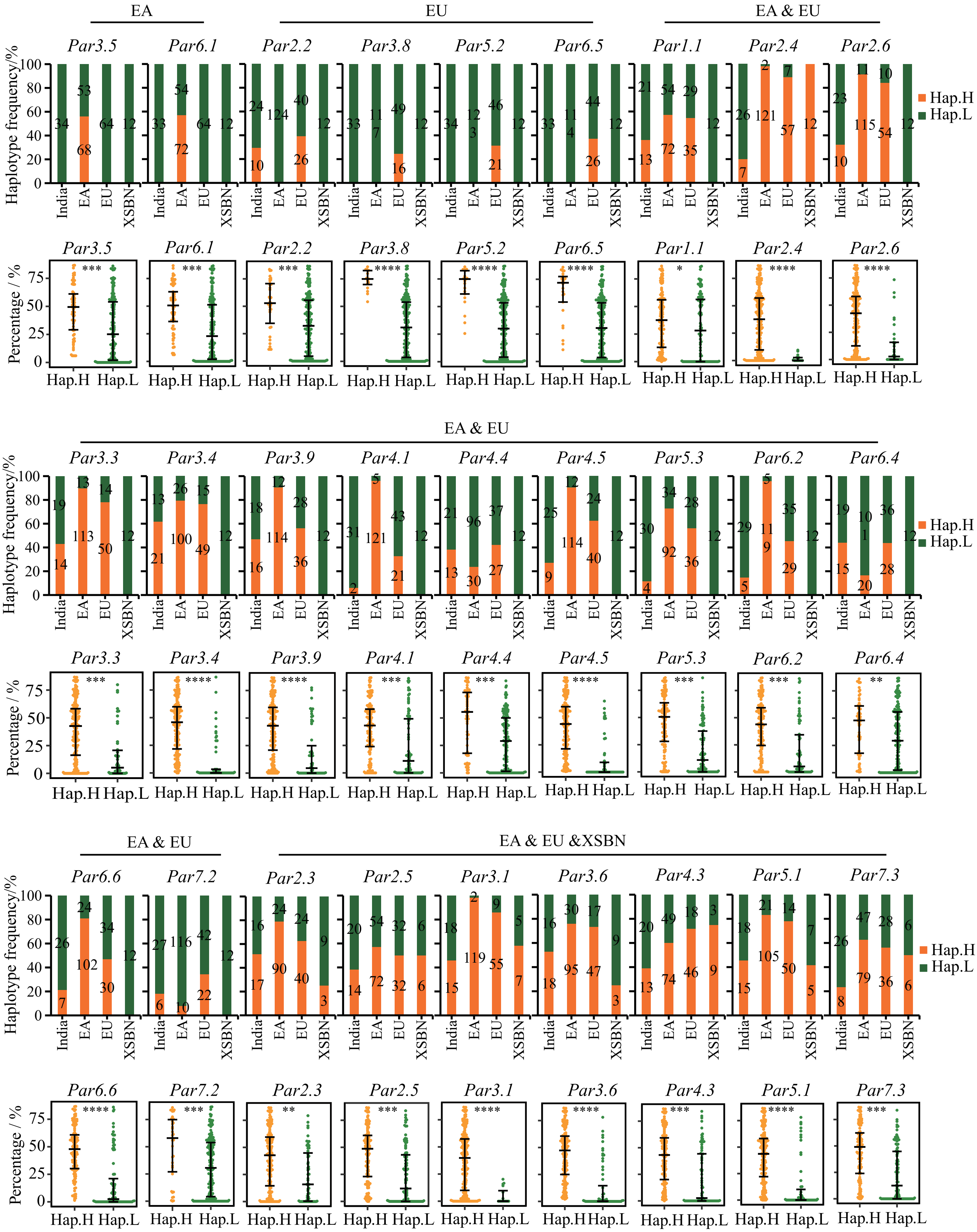

Fig. S4 Haplotype frequency of 27 GWAS signals in four groups among 236 GWAS populations. For each GWAS signal, Hap.H represents cucumbers with a high parthenocarpic fruit set rate, whereas Hap.L represents those with a low parthenocarpic fruit set rate. **, ***, and **** denote significant differences at $p < 0.01$, $p < 0.001$, and $p < 0.0001$, respectively, as determined by the Wilcoxon test.

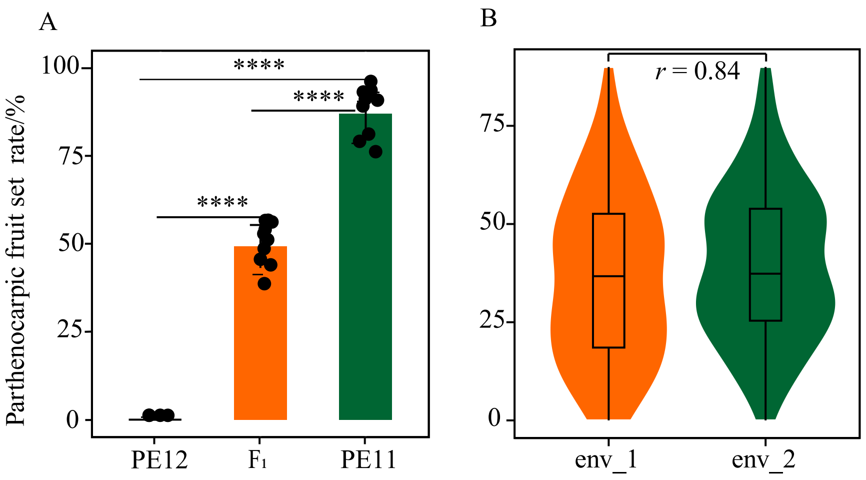

Fig. S5 Phenotypic data of the parental lines, their F_1 (**A**), and the mapping population $F_{2:3}$ across the two environments (**B**). **** indicates $p < 0.0001$,as determined via the Wilcoxon test.

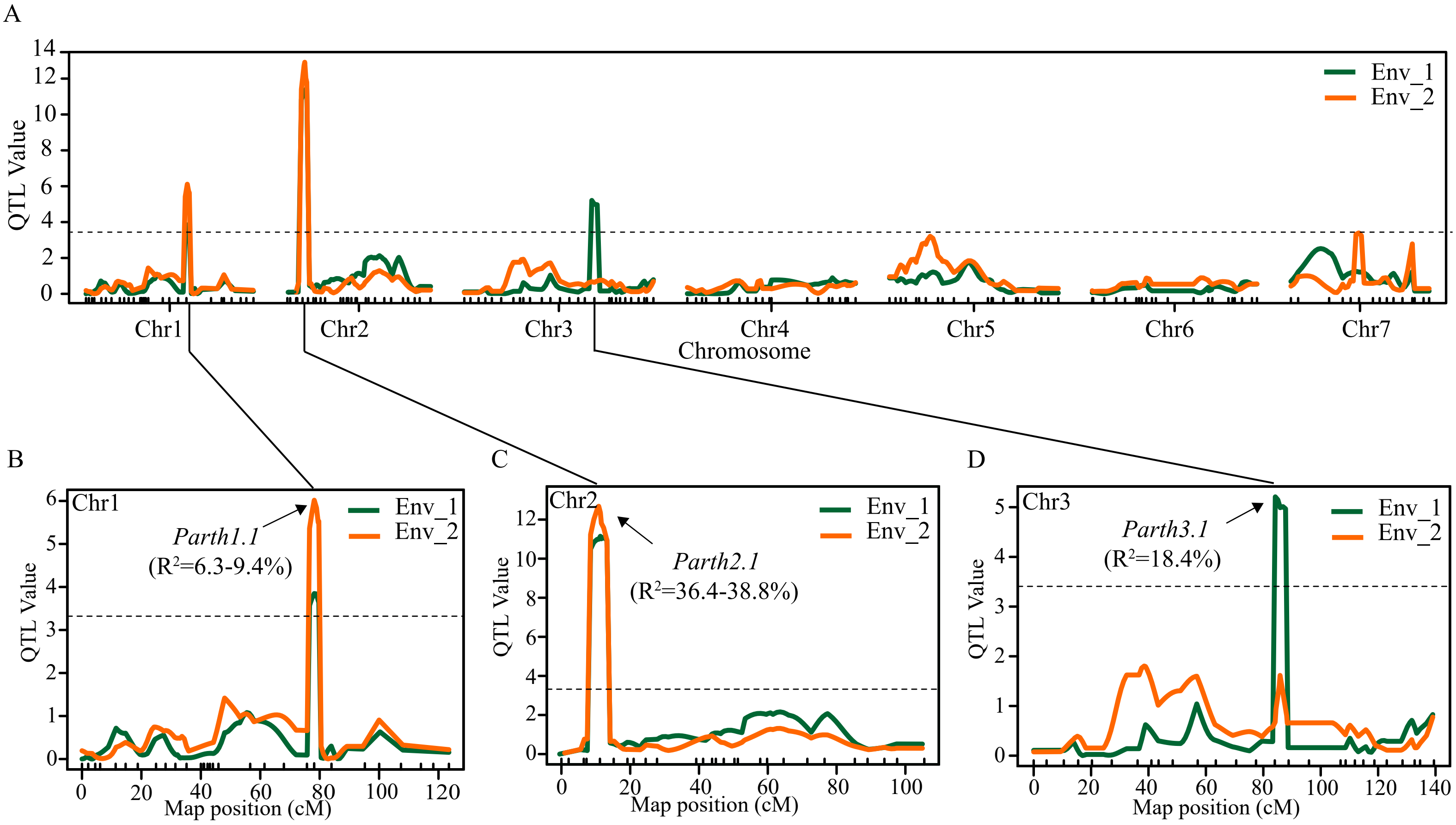

Fig. S6 QTL mapping for parthenocarpy using an $F_{2:3}$ population derived from a cross between PE11 and PE12. Three QTLs associated with parthenocarpy were detected on the chromosome of cucumber in two environments (**A**): *Parth1.1* (**B**), *Parth2.1* (**C**), and *Parth3.1* (**D**).

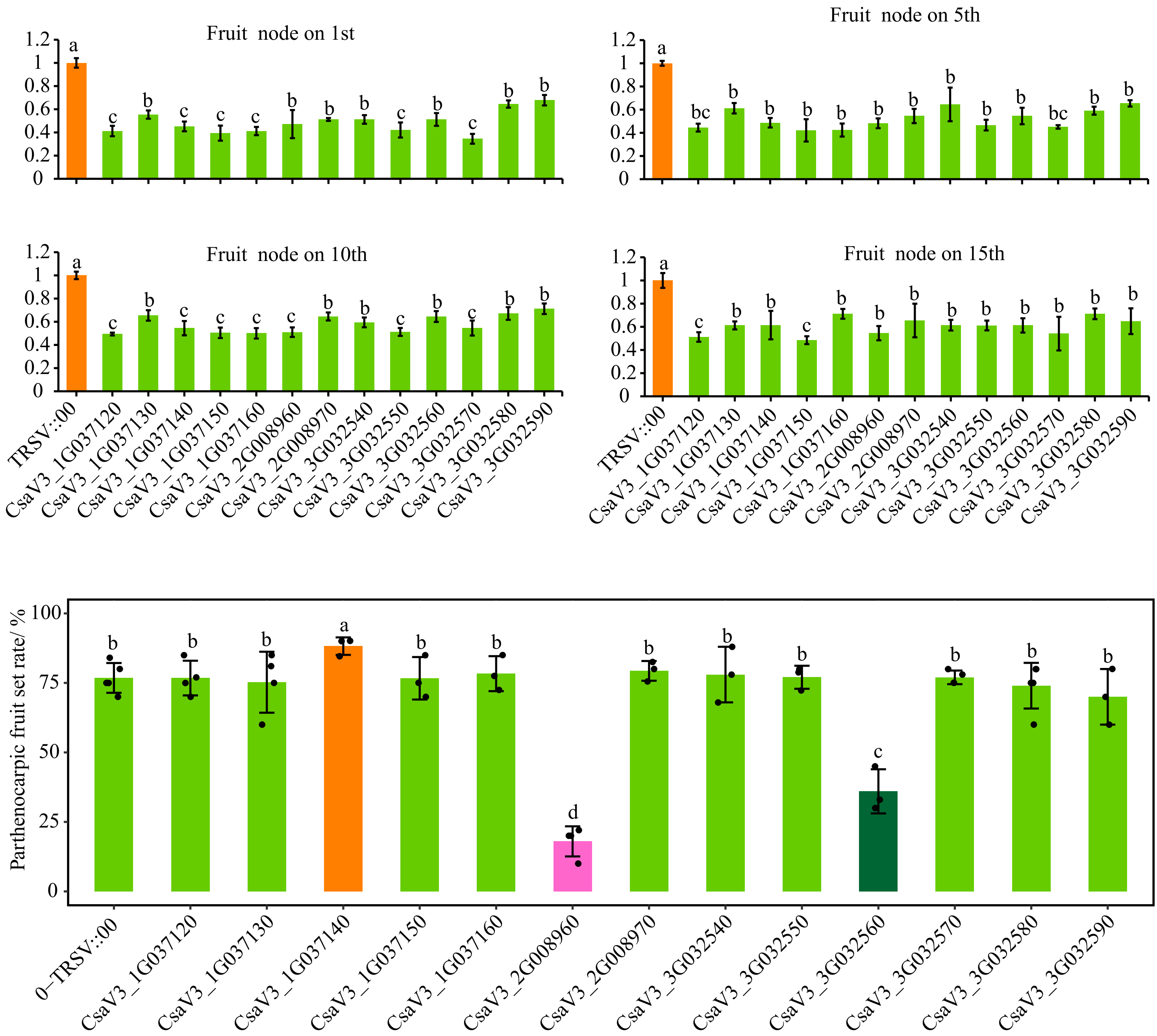

Fig. S 7 Validation of the 13 candidate genes associated with parthenocarpy via virus-induced gene silencing. The relative expression of 13 candidate genes in their VIGS lines was detected on the basis of parthenocarpic fruits collected at 2 days on the 1st (**A**), 5th (**B**), 10th (**C**), and 15th fruit nodes (**D**). **E** Comparison of the parthenocarpic fruit set rates of VIGS and wild-type plants. There were significant differences in parthenocarpic ability between the *CsaV3_1G037140*, *CsaV3_2G008960*, and *CsaV3_3G032560* VIGS lines and the wild-type cucumber line TRSV2::00. The data in **A**, **B**, **C**, **D**, and **E** are the means ± SDs, and significant differences at the $p < 0.05$ level, as determined by the Wilcoxon test, are indicated by different letters.

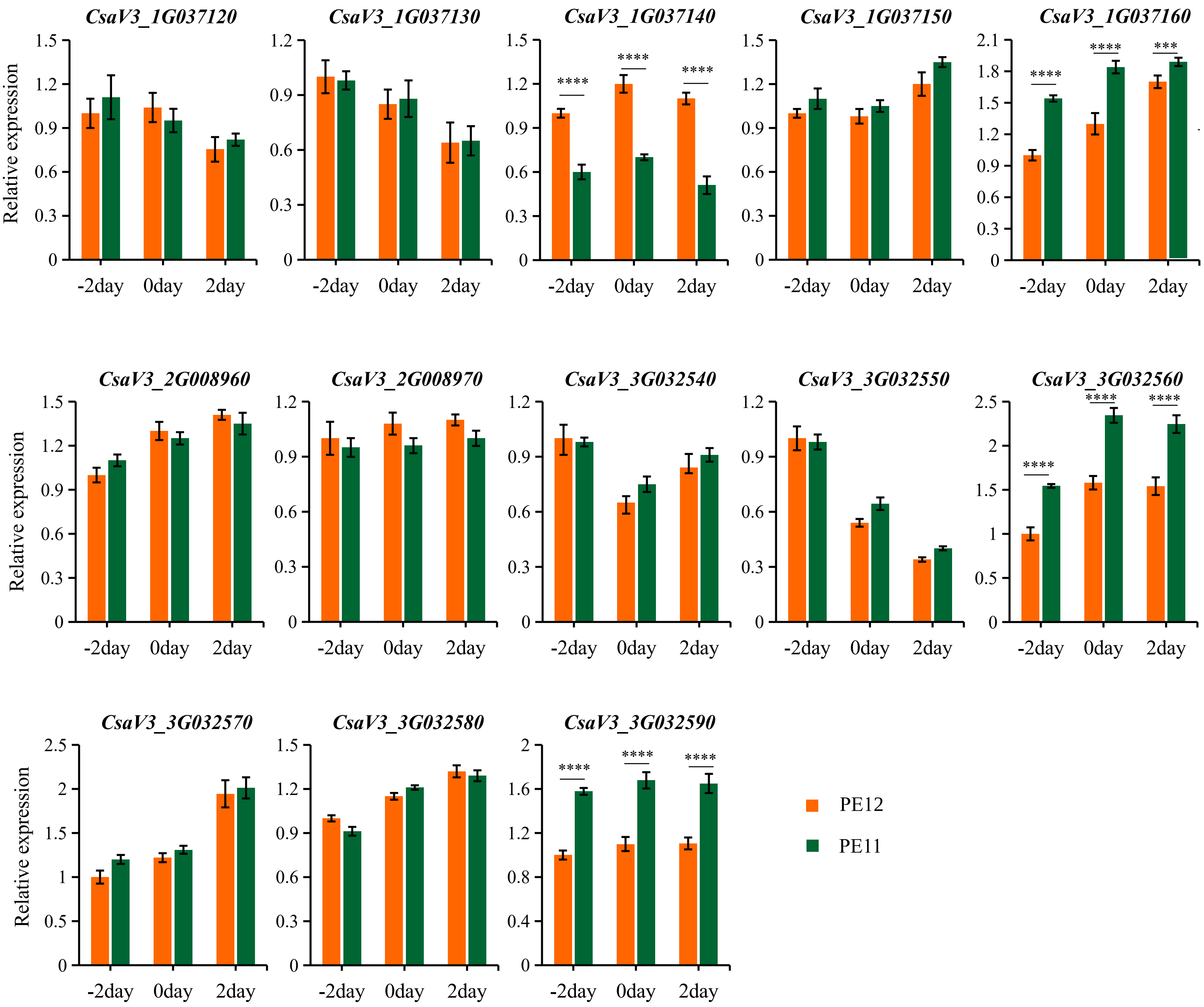

Fig. S8 Relative expression of 13 candidate GWAS genes from parthenocarpic fruits at -2 days, 0 days, and 2 days. **** indicates $p < 0.0001$, as determined by the Wilcoxon test.

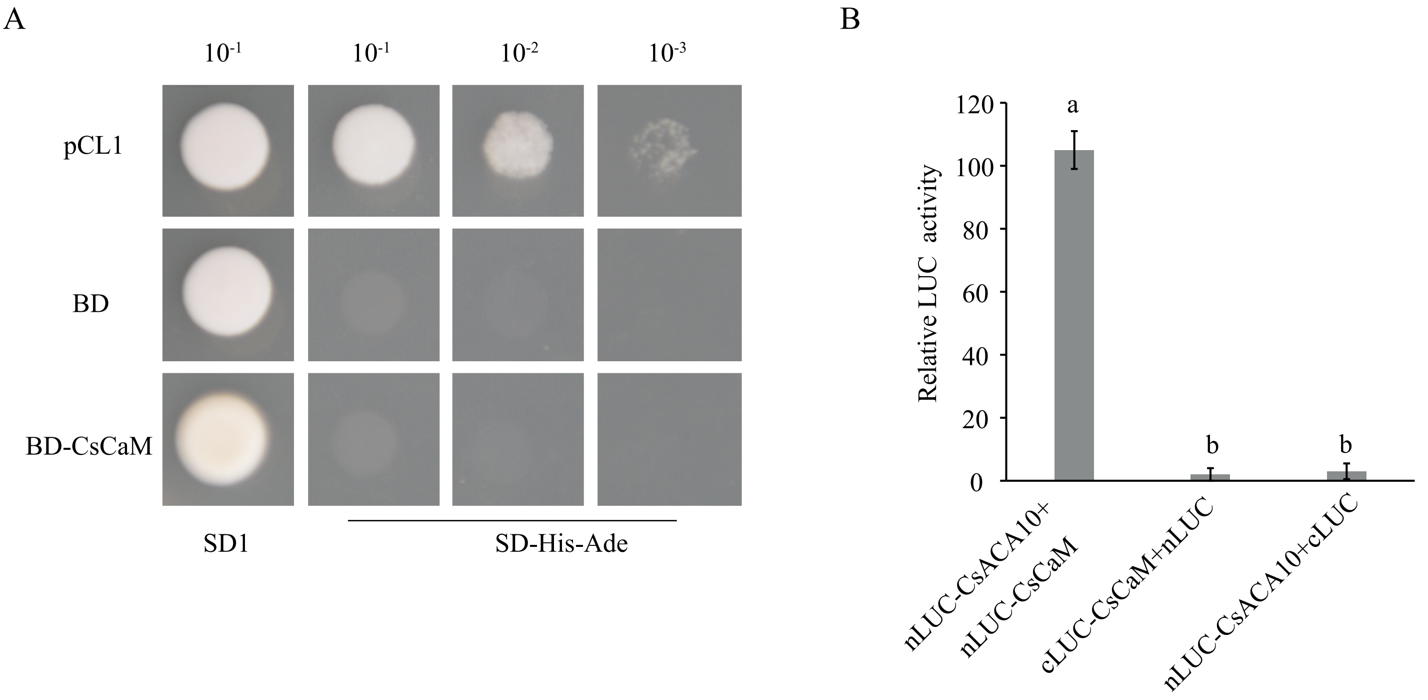

Figs. S9 Autoactivation verification assay of BD-CsCaM (A) and LUC enzyme activity assay (B). pCL1 and BD served as positive and negative controls in autoactivation verification assay, respectively; nLUC-CsACA10+cLUC and cLUC-CsCaM+nLUC were used as negative controls. The significant differences at the $p < 0.05$ level, as determined by the Wilcoxon test, are indicated by different letters.

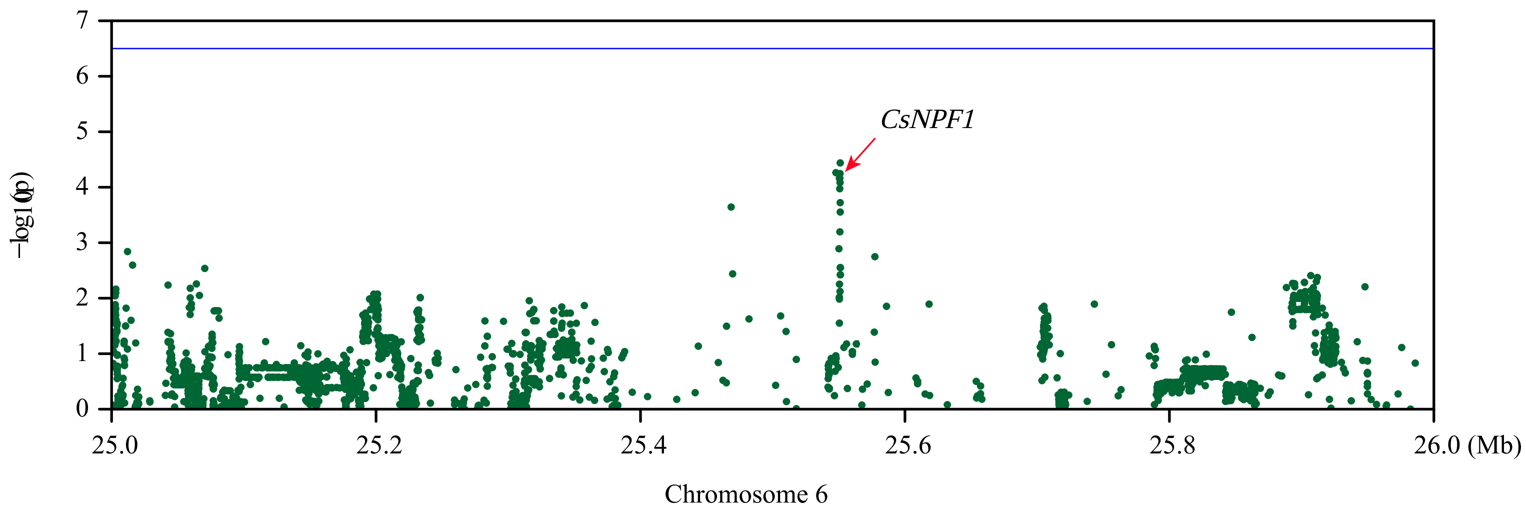

Figs. S10 GWAS signals associated with parthenocarpy near *CsNPF1*.

The blue line indicates significance threshold (p =1.5×10^{-7}) of GEMMA. The red arrow indicates p value of *CsNPF1*
